# A conserved 3′UTR short motif regulates gene expression in vertebrates

**DOI:** 10.1093/nar/gkaf1340

**Published:** 2026-01-08

**Authors:** Ana Eufrásio, Joana Machado, Joana Azevedo, Isabel Pereira-Castro, Alexandre Ferreira, Ana Moutinho, Filipe Henriques, Ana Jesus, Mafalda Araújo, Joana Tavares, Bruno Sousa, Bruno Cavadas, Iris Georgia Kessler, Joana Teixeira, Pedro Borges Pinto, José Bessa, Alexandra Moreira

**Affiliations:** Vertebrate Development and Regeneration Group, i3S - Universidade do Porto, 4200-135, Porto, Portugal; i3S – Instituto de Investigação e Inovação em Saúde, Universidade do Porto, 4200-135, Porto, Portugal; IBMC – Instituto de Biologia Molecular e Celular, Universidade do Porto, 4200-135, Porto, Portugal; ICBAS – Instituto de Ciências Biomédicas Abel Salazar, Universidade do Porto - Programa Doutoral em Biologia Molecular e Celular, 4050-313, Porto, Portugal; i3S – Instituto de Investigação e Inovação em Saúde, Universidade do Porto, 4200-135, Porto, Portugal; IBMC – Instituto de Biologia Molecular e Celular, Universidade do Porto, 4200-135, Porto, Portugal; Gene Regulation Group, i3S - Universidade do Porto, 4200-135, Porto, Portugal; Departamento de Química e Bioquímica, Faculdade de Ciências, Universidade de Lisboa, 1749-016, Lisboa, Portugal; i3S – Instituto de Investigação e Inovação em Saúde, Universidade do Porto, 4200-135, Porto, Portugal; IBMC – Instituto de Biologia Molecular e Celular, Universidade do Porto, 4200-135, Porto, Portugal; ICBAS – Instituto de Ciências Biomédicas Abel Salazar, Universidade do Porto - Programa Doutoral em Biologia Molecular e Celular, 4050-313, Porto, Portugal; Gene Regulation Group, i3S - Universidade do Porto, 4200-135, Porto, Portugal; i3S – Instituto de Investigação e Inovação em Saúde, Universidade do Porto, 4200-135, Porto, Portugal; IBMC – Instituto de Biologia Molecular e Celular, Universidade do Porto, 4200-135, Porto, Portugal; Gene Regulation Group, i3S - Universidade do Porto, 4200-135, Porto, Portugal; i3S – Instituto de Investigação e Inovação em Saúde, Universidade do Porto, 4200-135, Porto, Portugal; IBMC – Instituto de Biologia Molecular e Celular, Universidade do Porto, 4200-135, Porto, Portugal; Gene Regulation Group, i3S - Universidade do Porto, 4200-135, Porto, Portugal; i3S – Instituto de Investigação e Inovação em Saúde, Universidade do Porto, 4200-135, Porto, Portugal; IBMC – Instituto de Biologia Molecular e Celular, Universidade do Porto, 4200-135, Porto, Portugal; Gene Regulation Group, i3S - Universidade do Porto, 4200-135, Porto, Portugal; i3S – Instituto de Investigação e Inovação em Saúde, Universidade do Porto, 4200-135, Porto, Portugal; IBMC – Instituto de Biologia Molecular e Celular, Universidade do Porto, 4200-135, Porto, Portugal; Gene Regulation Group, i3S - Universidade do Porto, 4200-135, Porto, Portugal; i3S – Instituto de Investigação e Inovação em Saúde, Universidade do Porto, 4200-135, Porto, Portugal; IBMC – Instituto de Biologia Molecular e Celular, Universidade do Porto, 4200-135, Porto, Portugal; Gene Regulation Group, i3S - Universidade do Porto, 4200-135, Porto, Portugal; i3S – Instituto de Investigação e Inovação em Saúde, Universidade do Porto, 4200-135, Porto, Portugal; IBMC – Instituto de Biologia Molecular e Celular, Universidade do Porto, 4200-135, Porto, Portugal; Gene Regulation Group, i3S - Universidade do Porto, 4200-135, Porto, Portugal; GenoMed - Diagnósticos de Medicina Molecular SA, 1649-028, Lisboa, Portugal; i3S – Instituto de Investigação e Inovação em Saúde, Universidade do Porto, 4200-135, Porto, Portugal; IBMC – Instituto de Biologia Molecular e Celular, Universidade do Porto, 4200-135, Porto, Portugal; Gene Regulation Group, i3S - Universidade do Porto, 4200-135, Porto, Portugal; i3S – Instituto de Investigação e Inovação em Saúde, Universidade do Porto, 4200-135, Porto, Portugal; i3S – Instituto de Investigação e Inovação em Saúde, Universidade do Porto, 4200-135, Porto, Portugal; IBMC – Instituto de Biologia Molecular e Celular, Universidade do Porto, 4200-135, Porto, Portugal; Gene Regulation Group, i3S - Universidade do Porto, 4200-135, Porto, Portugal; Vertebrate Development and Regeneration Group, i3S - Universidade do Porto, 4200-135, Porto, Portugal; i3S – Instituto de Investigação e Inovação em Saúde, Universidade do Porto, 4200-135, Porto, Portugal; IBMC – Instituto de Biologia Molecular e Celular, Universidade do Porto, 4200-135, Porto, Portugal; IBMC – Instituto de Biologia Molecular e Celular, Universidade do Porto, 4200-135, Porto, Portugal; Gene Regulation Group, i3S - Universidade do Porto, 4200-135, Porto, Portugal; Vertebrate Development and Regeneration Group, i3S - Universidade do Porto, 4200-135, Porto, Portugal; i3S – Instituto de Investigação e Inovação em Saúde, Universidade do Porto, 4200-135, Porto, Portugal; IBMC – Instituto de Biologia Molecular e Celular, Universidade do Porto, 4200-135, Porto, Portugal; i3S – Instituto de Investigação e Inovação em Saúde, Universidade do Porto, 4200-135, Porto, Portugal; IBMC – Instituto de Biologia Molecular e Celular, Universidade do Porto, 4200-135, Porto, Portugal; Gene Regulation Group, i3S - Universidade do Porto, 4200-135, Porto, Portugal; ICBAS – Instituto de Ciências Biomédicas Abel Salazar, Universidade do Porto, 4050-313, Porto, Portugal

## Abstract

The messenger RNA (mRNA) 3' untranslated region (3'UTR) contains important regulatory sequences, including upstream sequence elements (USEs), which regulate gene expression. One well-characterised USE in the 3'UTR of the *Drosophila polo* gene affects adult fly phenotypes when disrupted. We have now identified a highly conserved sequence within this USE (DplUSE) in the 3'UTR of several vertebrate genes, including in zebrafish, mouse, and human genomes and show that DplUSE enhances gene expression in human cells and zebrafish embryos. We show that, in humans, DplUSE-containing genes are associated with congenital disease processes, and that disruption of DplUSE function impairs zebrafish development. We also found that HuR/ELAVL1, hnRNPC, and PTBP1/hnRNPI bind to *DplUSE* RNA and are required for its activity in a human cell line, suggesting a highly conserved mechanism across distantly related species. Our results indicate that PTBP1 has a global function in alternative polyadenylation, activating the selection of distal polyA sites and repressing intronic polyadenylation in DplUSE-containing genes while hnRNPC and HuR modulate their expression. Additionally, we found that a colon cancer-associated SNP in the *POU2AF2/C11orf53* 3'UTR creates an ectopic DplUSE site, increasing gene expression in zebrafish gut cells and in a human cell line. We have therefore identified a short 3'UTR motif present in diverse vertebrate genes that controls their expression through conserved RBPs interactions and is implicated in human disease.

## Graphical Abstract

**Graphical Abstract:**
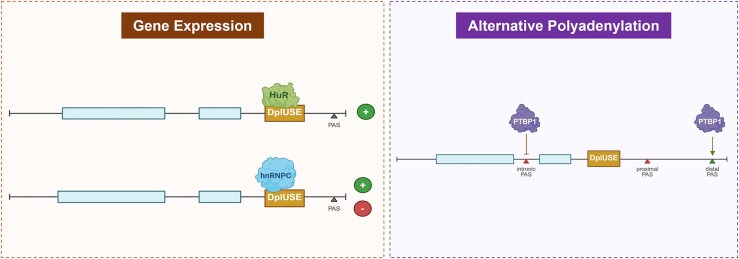

## Introduction

The 3′ untranslated region (3′UTR) of messenger RNA (mRNA) is defined by cleavage and polyadenylation of the pre-mRNA and has a central function in the regulation of gene expression. Regulation of expression is achieved by specific sequences in 3′UTRs that are recognised by RNA-binding proteins (RBPs) or targeted by miRNAs to control the fate of the mRNA (reviewed in [1–8]). Polyadenylation signals (PAS) in 3′UTRs are conserved among fish, flies, mammals, and worms [9]. Similar to mammals, the most common PAS in zebrafish (*Danio rerio*) is the canonical AAUAAA, which, together with GU/U-rich downstream sequences, is present in 62.3% of pre-mRNAs [10]. However, about half of pre-mRNAs contain a non-canonical PAS [11, 12], despite being efficiently cleaved and polyadenylated. Usage of non-canonical PAS is enhanced and fine-tuned by the presence of auxiliary *cis*-elements in the pre-mRNA, such as the upstream sequence elements (USEs) that are localised upstream of the PAS. Therefore, USEs are fundamental for the proper control of gene activity. USEs have been characterised in many human genes as sequences that activate PAS selection and 3′ end formation efficiency by acting as an extra platform for the recruitment of *trans*-acting factors, such as RBPs, which have important functions in diseases [5, 13–30] (Table 1). USEs have also been identified in other species such as the fruit fly (*Drosophila melanogaster*). In the 3′UTR of the *Drosophila* gene *polo*, which codes for a key cell-cycle kinase [31, 32], a highly conserved 28-nucleotide USE activates the non-canonical proximal PAS (AUUAAA), which enhances Polo protein levels at kinetochores [15]. Previous bioinformatic analysis has shown that a short region of this USE is conserved between the 50-million-year-distant *Drosophila melanogaster* and *Drosophila albomicans* [15], suggesting a conserved regulatory function for this non-coding sequence. We have also shown that 5.2% of *D. melanogaster* and 2.7% of human 3′UTRs contain this USE upstream of an AUUAAA non-canonical PAS [15]. While *polo*’s USE is a member of the USE family, for simplicity, we have named the most conserved sequence (TTGTTTT) within the *polo* USE, DplUSE (*Drosophila polo* USE). A sequence similar to DplUSE, identified in the 3′UTR of the human gene *prothrombin F2*, regulates gene activity and has a function in human disease [33, 34] (Table 1). All these results suggest that the DplUSE sequence may play a highly conserved role in the regulation of vertebrate gene expression.

Here, we show that the DplUSE sequence is present in the 3′UTR of several genes in zebrafish, mouse, and human genomes, suggesting a conserved role of the DplUSE sequence in gene regulation in vertebrates. To understand DplUSE function, we have performed *in vivo* reporter assays in zebrafish, using a part of the 3′UTR of the mRNA for *Drosophila polo* that includes the DplUSE sequence, linked to a GFP reporter gene. We find that DplUSE increases the expression of GFP in several zebrafish tissues. Additionally, we demonstrate that microinjection of *DplUSE* RNA in one-cell-stage zebrafish embryos leads to several abnormalities during embryonic development, possibly by sequestering the specific RBPs that recognise endogenous DplUSEs. These results suggest that the molecular machinery that operates on the DplUSE is required for the proper early embryonic development of zebrafish. To better understand the molecular mechanism involved in DplUSE function, we explored the binding of candidate RBPs to the *DplUSE* RNA sequence. We show that HuR/ELAVL1 [35], hnRNP C [21, 36], and PTBP1, the orthologue RBP that is required for DplUSE function in *Drosophila* [37–40], bind to *DplUSE* RNA. In addition, we show that HuR and hnRNP C modulate the expression of DplUSE-containing genes in HeLa cells, and that PTBP1 activates distal polyA site selection and represses intronic polyadenylation, confirming their role in DplUSE function. Finally, we searched for single nucleotide polymorphisms (SNPs) that might affect the DplUSE sequence and are associated with human disease. We found that the rs3087967 SNP, associated with malignant tumour of the colon, is located in the 3′UTR of the oncogene *POU2AF2/C11orf53* [41–43] and creates an ectopic DplUSE consensus, causing a gain of function of the mRNA *in vivo* in the zebrafish gut and in a human cell line, suggesting its involvement in colon cancer.

Collectively, we provide *in vivo* evidence that DplUSE functions as a regulator of gene expression in zebrafish, demonstrate its conserved RBPs-mediated mechanism of action in zebrafish and humans, and implicate the DplUSE consensus sequence in human disease.

## Materials and methods

### Bioinformatic analysis

#### Selection of 3′UTR sequences

The NCBI RefSeq transcripts and the respective genes of *Danio rerio* (assembly danRer10), *Mus musculus* (assembly mm10), and *Homo sapiens* (assembly hg19) genomes were retrieved from UCSC browser [44]. The 3′UTR regions were selected and queried for the presence of the DplUSE sequence (TTGTTTT) followed by the sequence of the *Drosophila melanogaster’s polo* proximal PAS (ATTAAA) spaced at a maximum distance of 450 bp, using an R-based script. Subsequently, using these references, we obtain the respective genes applying BioMart (https://www.ensembl.org/biomart/martview). The zebrafish and mouse genes were converted to the human orthologs using the BioMart web tool [45], which functions based on homology mapping between different species interlinked in Ensembl database.

The Venn diagrams were constructed using BioVenn. The Gene Ontology terms from human, zebrafish, and mouse DplUSE-containing gene lists were obtained by GO Enrichment Analysis using Panther tools [46], and the results presented in the figures are from Biological Processes terms. The results obtained are based on the relative enrichment to the set of all protein-coding genes. Only GO terms related to biological processes with a false discovery rate (FDR) < 0.05 were considered. The terms were ordered by fold enrichment (FE) and then by FDR.

#### Frequency of regions containing DplUSE upstream of ATTAAA in human, zebrafish, and mouse

The median length of the 3′UTR in human genes is ~600 nucleotides for single-UTR genes and ∼2300 nucleotides for multi-UTR genes [47]. We analysed the distances between the DplUSE (TTGTTTT) and the non-canonical PAS (ATTAAA) in the human, zebrafish, and mouse genomes. Only distances up to 3000 base pairs (bp) were considered. Then, we identified the genomic positions of the sequences using the previous R-based script. For each TTGTTTT sequence, the distance to the nearest ATTAAA was calculated. The distances were grouped into intervals of 50 bp, and the frequency within each interval was counted.

#### Identification of disease-associated SNPs overlapping with the DplUSE motif

After obtaining the coordinates for the DplUSE sequence using the R-based script previously mentioned, we extracted all the disease variations from DisGeNet datasets (https://www.disgenet.org/home/) and the respective coordinates. The disease data were sourced from human-curated information, combining data from UniProt, ClinVar, Orphanet, the GWAS Catalogue, and CTD, using the Python programming language. Then, using BEDtools intersect (v.2.27), we intercepted both datasets in order to obtain the disease-associated SNPs that overlapped with DpIUSE motif.

#### Gene–disease association and 3′UTR sequence conservation analysis

The gene–disease association for each gene was retrieved from DisGeNET using human-curated datasets that integrate information from UniProt, ClinVar, Orphanet, the GWAS Catalogue, and CTD. The percentage of disease-associated genes was calculated for DplUSE gene lists (31 and 2110) and compared to a control that is the percentage of genes linked to the same disease for the total pool of genes present in the DisGeNET database.

The 3′UTR coordinate regions were obtained from USCS genome browser and the phyloP values for each nucleotide from 100 vertebrate comparison dataset tracks were obtained. This dataset represents multiple alignments of 100 vertebrate species and the measurement of evolutionary conservation using phyloP values. phyloP values serve as a metric for evolutionary conservation at individual alignment sites. Positive scores indicate higher conservation, while negative values suggest lower conservation, relative to the evolution predicted under neutral drift.

### Oligonucleotides, antibodies, and siRNAs

The oligonucleotides, antibodies, and siRNAs used in this work are listed in Supplementary Table S1.

### Plasmid constructs

For the *in vitro* transcription plasmids for zebrafish microinjection, pCS2-GFP-DplUSE and pCS2-GFP-DplUSEmt contain the DplUSE and DplUSEmt sequences and the remaining polo’s 3′UTR up to the PAS ATTAAA (included). This DNA fragment was subcloned downstream of the reporter gene GFP in *Xho*I and *Kpn*I restriction sites of pCS2-GFP vector (Addgene plasmid #105937), removing the SV40 late PAS from the vector.

For the generation of the pUC19miniTOL-GFP-DplUSE and pUC19miniTOL-GFP-DplUSEmt plasmids, the DplUSE and DplUSEmt sequences were subcloned downstream of the GFP reporter gene into the *Sal*I and *Kpn*I restriction sites of the pUC19miniTOL vector [48], to allow efficient genomic integration in zebrafish embryos when co-injected with Tol2 transposase. pUC19miniTOL-GFP-DplUSE and pUC19miniTOL-GFP-DplUSEmt plasmids were also used for the human cell transfection experiments.

For the generation of the control pUC19miniTOL-GFP-ΔDplUSE plasmid for human cell line transfection, the 150 bp fragment of *polo’s* 3′UTR, where the DplUSE sequence is included, was deleted from the pUC19miniTOL-GFP-DplUSE by inverse PCR using the InvPCR_3UTRpolo_F1 phosphorylated primer and the InvPCR_3UTRpolo_R primer (Supplementary Table S1). The InvPCR_3UTRpolo_F1 primer was phosphorylated using the T4 Polynucleotide Kinase (NEB) and the PCR product was re-ligated using the T4 DNA ligase (Thermo Fisher Scientific).

For the generation of the control pUC19miniTOL-GFP-MCS plasmid for human cell line transfection, the 150 bp fragment of *polo’s* 3′UTR of the pUC19miniTOL-GFP-DplUSE plasmid was substituted by the 103 bp DNA fragment of the pcDNA3.1 plasmid (Thermo Fisher) polylinker (MCS). To delete the DplUSE sequence, an inverse PCR using the InvPCR_3UTRpolo_F2 and InvPCR_3UTRpolo_NheI_R primers (Supplementary Table S1) was performed. The PCR product was then digested with *NheI* restriction enzyme (NEB). The MCS from the pcDNA3.1 plasmid was isolated by PCR amplification using the T7_promoter_F and the BGH_R primers (Supplementary Table S1). The PCR product was digested with *ApaI* restriction enzyme (NEB), and blunt ends were produced using the DNA Polymerase I, Large (Klenow) Fragment (NEB), following digestion with *NheI* restriction enzyme (NEB) to subclone the MCS into pUC19miniTOL-GFP-MCS.

To produce the plasmids to assess the *C11orf53* risk and non-risk variants in zebrafish and in a human cell line, the 3′UTR of the POU2AF2/C11orf53 gene was subcloned into *Xho*I and *Kpn*I restriction sites of pUC19miniTOL-GFP-DplUSE to obtain the plasmid pUC19miniTOL-GFP-DplUSE-POU2AF2/C11orf53 non-risk variant. Next, we used site-directed mutagenesis to obtain pUC19miniTOL-GFP-DplUSE-POU2AF2/C11orf53 risk variant (see Supplementary Table S1).

For the *in vitro* experiments, DplUSE and DplUSE mutant plasmids depicted in Fig. 4B were constructed by subcloning DplUSE and DplUSEmt oligonucleotides into the SmaI restricition site of pGEM-7Zf(+) [49].

For the luciferase assays in HEK293 cells, the pLuc-DplUSE non-risk variant and pLuc-DplUSE risk variant luciferase reporter plasmids were made by amplification of the non-risk variant and the risk variant DplUSE sequences of POUF2AF2/C11orf53 using the template plasmids pUC19miniTOL-DplUSE-POU2AF2/C11orf53–non-risk variant and pUC19miniTOL-DplUSE-POU2AF2/C11orf53–risk variant, and forward (FW_useORF) and reverse (RV_useORF) primers with *Xho*l and *Kpn*I restriction sites, respectively (Supplementary Table S1). The PCR products and the pLuc plasmid [50] were digested with *Xho*l and *Kpn*I restriction enzymes (NEB) to clone the non-risk and risk variants downstream of the *luc* gene on the pLuc plasmid.

### *In vitro* transcription

For synthesis of capped mRNAs to microinject into zebrafish embryos, the template plasmids were initially linearized with appropriate restriction enzymes. *GFP-DplUSE* and *GFP-DplUSEmt* pre-mRNAs were synthesized by *in vitro* transcription using as templates the pCS2-GFP-DplUSE and pCS2-GFP-DplUSEmt constructs, linearized with *Kpn*I-HF restriction enzyme, and SP6 RNA polymerase to transcribe.

DplUSE and DplUSE mutants’ pre-mRNAs depicted in Fig. 4 were synthesized by *in vitro* transcription, using DplUSE and DplUSE mutant constructs as templates, linearized with *Bam*HI or ClaI. *In vitro* transcription was performed as previously described [40], with minor modifications, by incubating 1 μg of linearized plasmids with 1× transcription buffer (Roche Applied Science), 10 mM DTT, 28 U of RNAguard™ Ribonuclease Inhibitor, 0.1 mM GTP, 0.5 mM CTP, 0.05 mM ATP and UTP, 1 mM Cap Analogue (Ambion), 8 μCi of α-^32^P-[ATP] and α-^32^P-[UTP], and 20 U of T7 RNA Polymerase or SP6 RNA Polymerase (Roche Applied Sciences). The reaction was incubated for 1 h at 37°C. The DNA was digested by adding 5 U of DNase I and incubating at 37°C for 15 min. The transcripts were purified using Illustra ProbeQuant^TM^ G-50 Micro Columns (Cytiva).

### UV crosslinking assays

UV crosslinking assays were performed as previously described [40] with minor modifications. Briefly, a mixture containing 2 mM ethylenediaminetetraacetic acid (EDTA), 1 mM ATP, 20 mM CP, 11.5 U RNAguard™ Ribonuclease Inhibitor, 2.5% (w/v) PVA, and 40 μg/ml of tRNA was incubated with 100 cps of radiolabelled RNA and 5 μl of HeLa cell nuclear extracts. Reactions were incubated for 10 min at 30°C then 1 μl of 2.5 μg/ml tRNA was added. For competition assays 1, 50, and 150 pmoles of unlabelled competitor RNAs were added together with labelled RNA. The reaction mixtures were irradiated twice with 96 × 10^4^ μJ/cm^3^ of UV light for 3 min, using the Hoefer UVC 500 Ultraviolet Crosslinker (GE Healthcare Life Sciences). RNase A was added to the samples, for 30 min at 37°C, to degrade unprotected RNA. Samples were boiled at 95°C for 5 min in a 2× SDS gel-loading buffer in order to denature proteins and separated by electrophoresis in a 10% sodium dodecyl sulphate–polyacrylamide gel electrophoresis (SDS–PAGE). The gel was fixed with 10% (v/v) acetic acid and 10% (v/v) glycerol solution for 30 min at room temperature, and then dried at 80°C under vacuum for 2 h. The radiolabelled protein bands were visualized by autoradiography.

### Immunoprecipitations

For immunoprecipitation of UV crosslinked proteins with monoclonal antibodies 4F4 anti-hnRNP C (mouse) (kind gift from Gideon Dreyfuss, Howard Hughes Medical Institute, University of Pennsylvania School of Medicine) and HuR (19F12, Thermo) 400 μl of 10% (v/v) protein A Sepharose CL-4B beads (GE Healthcare Life Sciences) in IP-2 buffer [50 mM Tris pH 7.9, 50 mM NaCl, 0.1% (v/v) NP-40] was incubated with 40 μg of rabbit anti-mouse antibody (DakoCytomation) in a vertical wheel for 90 min at 4°C. The beads were washed three times with ice-cold IP-2 buffer. One UV crosslinking reaction and 2 μl of anti-hnRNP C or HuR were added to the beads and the mixture was rotated for 1 h at 4°C. The beads were subsequently washed three times and dried. For PTBP1 immunoprecipitation, a rabbit anti-PTBP1 serum was used (generous gift from Chris W. J. Smith, Department of Biochemistry, University of Cambridge). To 100 μl of 50% (v/v) protein A sepharose-PBS, 20 μl of anti-PTBP1 and 600 μl of PBS were added, and the mix was incubated for 1 h at 4°C with gentle mixing. The beads were then washed with PBS and a UV crosslinking reaction was added and incubated overnight at 4°C. After incubation, the beads were washed two times with 800 μl of binding buffer I [20 mM Hepes pH 7.9, 150 mM NaCl, 0.05% (v/v) Triton X-100] and then two times with binding buffer II [20 mM Hepes pH 7.9, 150 mM NaCl, 1% (v/v) Triton X-100]. As a non-specific control, an immunoprecipitation was performed using a rabbit pre-immune (PI) serum. Twenty microlitres of 2× SDS gel-loading buffer were added to the beads, and the proteins were denatured at 95°C for 5 min and separated by gel electrophoresis in a 10% SDS–PAGE. The gel was fixed and dried and the radiolabelled protein bands were visualized by autoradiography.

### Zebrafish husbandry and embryo culture

Adult wild-type Tuebingen (TU) zebrafish (*Danio rerio*) were maintained in a recirculating system under conditions approved by the i3S Animal Welfare and Ethics Committee and the Portuguese National Authority for Animal Health (DGAV). Fertilized eggs were kept at 28°C in E3 medium supplemented with 0.001% 1-phenyl-2-thiourea to prevent pigmentation development [51].

### Microinjection procedures, zebrafish GFP-DplUSE transgenic line establishment, GFP screening, and quantification

**Figure 1.**
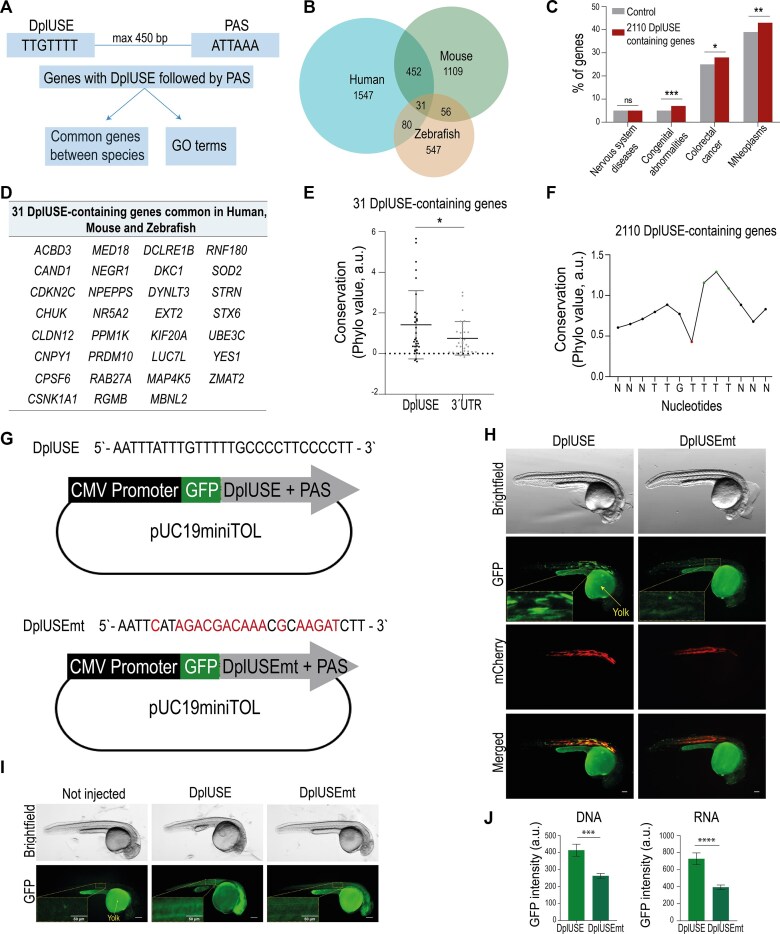
The DplUSE sequence is conserved across human, zebrafish, and mouse genes and increases GFP expression in zebrafish. (**A**) Workflow used to obtain the Gene Ontology (GO) terms related to biological processes of the common DplUSE-containing genes between the human and zebrafish transcriptomes obtained using an R-based bioinformatic script, which identified all the genes that contained, in the 3′UTR, the most conserved region of the USE sequence, DplUSE (TTGTTTT) located upstream of polo’s polyadenylation signal (ATTAAA), at a maximum separating distance of 450 base pairs (bp). (**B**) Venn diagram representing the number of DplUSE-containing genes found in human (2110), zebrafish (714), and mouse (1648) genomes. Eighty DplUSE-containing genes are common between humans and zebrafish, while 31 are common between the 3 species. (**C**) Representative graph showing the percentage of the 2110 DplUSE-containing human genes that are associated with the respective disease (DisGenet data source). The control represents the percentage of genes associated with the specific trait/disease in the DisGeNET database. (**D**) The 31 DplUSE-containing genes that are common between the 3 species: human, mouse, and zebrafish. (**E**) Representative graph of the average value of the extracted conservation values from each nucleotide within the DplUSE and 3′UTR sequence for each one of the 31 orthologous genes (data source: 100 vertebrates). (**F**) Representative graph showing the average conservation value for each nucleotide within the 2110 human DplUSE-containing genes. (**G**) Schematic representation of pUC19miniTOL-GFP-DplUSE and pUC19miniTOL-GFP-DplUSEmt plasmids used to microinject one-cell stage zebrafish embryos. The black boxes correspond to the cytomegalovirus (CMV) promoter region, the green boxes correspond to the GFP reporter gene, and the grey boxes represent the DplUSE sequence, or the DplUSE sequence mutated in 17 nucleotides, followed by the polyadenylation signal (PAS)—ATTAAA. (**H**) Representative images from 24 hpf zebrafish embryos microinjected with pUC19miniTOL-GFP-DplUSE/ pUC19miniTOL-GFP-DplUSEmt with Tol2 mRNA and mylz:mCherry vector. (**I**) Representative images from 24 hpf zebrafish embryos microinjected with *GFP-DplUSE* RNA and *GFP-DplUSEmt* RNA. (**J**) Representative graph for the GFP expression quantification from microinjected embryos with pUC19miniTOL-GFP-DplUSE and pUC19miniTOL-GFP-DplUSEmt (left); representative graph for the GFP expression quantification from microinjected embryos with *GFP-DplUSE* and *GFP-DplUSEmt* RNAs. The dashed lines represent the regions where GFP was quantified. Statistical significance was determined by χ2 test with Fisher correction or by two-tailed unpaired *t*-test. ***P < .001; **P < .01; *P < .05; ns *P *> .05. Images were acquired with Leica M205 (scale bar = 100 μm) and the GFP expression was quantified in at least 25 embryos for each condition.

**Figure 2.**
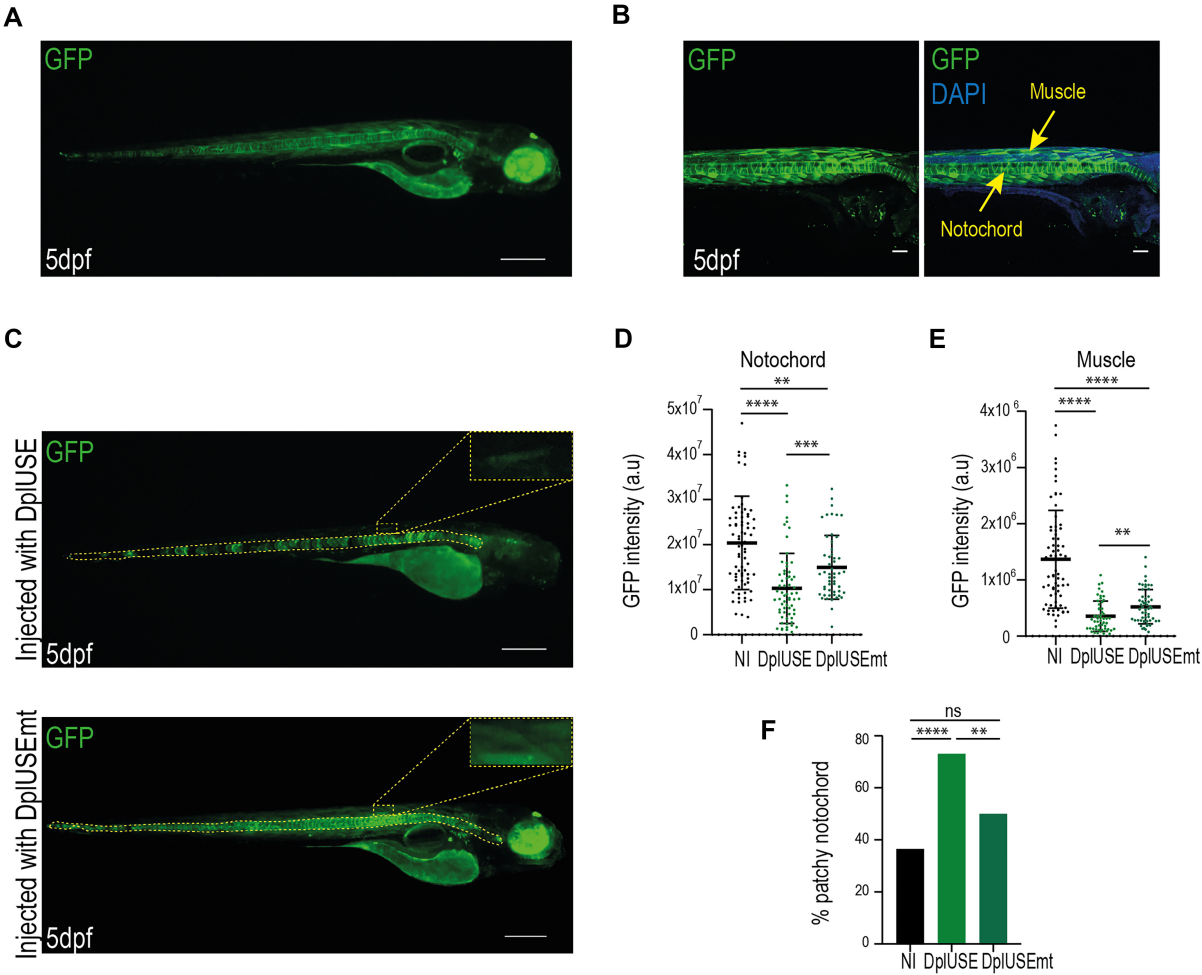
Microinjection of *DplUSE* RNA causes a decrease in GFP expression in transgenic GFP-DplUSE animals. (**A**) Representative image of a 5 dpf GFP-DplUSE transgenic line. (**B**) Confocal images of transgenic GFP-DplUSE zebrafish, stained with the nuclear marker DAPI, showing GFP expression in the notochord and muscle (scale bar = 10 μm). (**C**) Representative image of GFP expression in transgenic GFP-DplUSE zebrafish embryos microinjected with *DplUSE* RNA or *DplUSEmt* RNA at 5 dpf. Images were acquired using Leica M205. (**D**) Quantification of GFP expression in notochord (*n* = 75 Non Injected (NI); *n* = 64 DplUSE; *n* = 54 DplUSEmt) and (**E**) in muscle (*n* = 70 NI; *n* = 53 DplUSE; *n* = 54 DplUSEmt). (**F**) Quantification of the percentage of transgenic GFP-DplUSE embryos that showed a patchy expression of GFP in the notochord (36.5%, *n* = 73 NI; 73%, *n* = 69 DplUSE; 50%, *n* = 59 DplUSEmt). The dashed lines represent the regions where GFP was quantified. Statistical significance was determined by χ2 test with Fisher correction or by two-tailed unpaired t-test. ***P < .001; **P < .01; *P < .05; ns *P *> .05. (scale bar = 100 μm).

One-cell stage AB WT embryos were microinjected with 3 nl containing 25 ng/µl *Tol2* transposase mRNA and 25 ng/µl phenol/chloroform-purified reporter vectors: pUC19miniTOL-GFP-DplUSE, pUC19miniTOL-GFP-DplUSEmt, and mylz:mCherry, the latter being used as internal control of transgenesis, driving mCherry expression specifically in muscle fibres of the zebrafish. Embryos were screened at 24 h post-fertilization (hpf) for GFP expression, using a fluorescence stereomicroscope. The images were acquired using Leica M205 and analysed using ImageJ software [52]. For the GFP-RNA microinjection, one-cell stage AB WT embryos were microinjected with 3 nl containing 300 ng/µl *GFP-DplUSE* and *GFP-DplUSEmt* RNAs. Embryos were screened and quantified at 24 hpf for GFP expression as described before. Generation of the GFP-DplUSE zebrafish transgenic line was performed using the *Tol2* transposon system. One-cell stage AB wild-type embryos were microinjected with 3 nl containing 25 ng/µl *Tol2* transposase mRNA and 25 ng/µl phenol/chloroform-purified reporter vector (pUC19miniTOL-GFP-DplUSE). Embryos were screened at 48 hpf for GFP expression, using a fluorescence stereomicroscope, and raised until adulthood. Potential founders were then crossed with WT zebrafish, and the progeny with consistent GFP expression were selected and raised until adulthood, establishing in this way the GFP-DplUSE transgenic line. For the DplUSE-RNA microinjection, one-cell stage AB WT embryos were microinjected with 3 nl containing 300 ng/µl *DplUSE* and *DplUSEmt* RNAs. Embryos were screened and quantified at 24 hpf and 5 days post-fertilization (dpf) for GFP expression as described before. For GFP quantification in Fig. 1H and I, a region was selected in the embryo’s body, excluding the yolk sac due to its high autofluorescence. These regions are marked by dashed boxes. Similarly, for Fig. 2C and Supplementary Fig. 2A, regions were defined for the muscle and notochord, as indicated by dashed lines. A randomly chosen debris-free area outside the embryo was used to measure background fluorescence. GFP fluorescence was calculated as integrated density of the selected region − (area of selected region × mean fluorescence of background). All quantifications were performed in ImageJ.

For the assessment of the impact of the rs3087967 risk variant, we performed an *in vivo* mosaic transgenesis assay. One-cell stage AB WT embryos were microinjected with 3 nl containing 25 ng/µl Tol2 transposase mRNA and 25 ng/µl phenol/chloroform-purified reporter vectors (pUC19miniTOL-GFP-DplUSE-POU2AF2/C11orf53—non-risk variant and pUC19miniTOL-GFP-DplUSE-POU2AF2/C11orf53 risk variant and mylz:mCherry). Embryos were raised until they reached the 4 dpf larval stage, fixed, and stained with DAPI [PUREBLU™ DAPI (1351303; Bio-Rad)]. The images were acquired using SP5II confocal microscope.

### Immunohistochemistry

Transgenic GFP-DplUSE zebrafish embryos were fixed in 4% formaldehyde in PBS 1× overnight at 4°C, and then washed in PBS-T (0.1% Triton X-100 in PBS 1×), permeabilized in PBS-T 0.5%, rewashed, and incubated with DAPI (1:1000) (Invitrogen) diluted in PBS-T 0.1%, for 4 h. Embryos were extensively washed and mounted in microscopic slides in 50% glycerol in PBS 1×. Images were acquired using Leica SP5II confocal microscope and analysed in ImageJ. *DplUSE* and *DplUSEmt* RNA-microinjected embryos were fixed and washed as described before. For permeabilization, the embryos were incubated with 100% cold MeOH and blocked with *in situ* hybridization buffer. The embryos were then incubated with anti-cleaved caspase 3 pAb (1:100) [Anti-caspase-3, cleaved (Ab-2) Rabbit pAB (PC679; Merck)] followed by a wash and a second incubation with anti-rabbit 568. Finally, the embryos were washed, the images were acquired in Leica M205, and analysed in ImageJ.

pUC19miniTOL-GFP-DplUSE normal variant and pUC19miniTOL-GFP-DplUSE risk variant microinjected embryos were fixed, washed, and incubated with DAPI as described before. Images were acquired using Leica SP5II confocal microscope and analysed in IMARIS software [Imaris (RRID:SCR_007370)].

### RNA extraction, cDNA synthesis, and RT-qPCR

Total RNA was extracted using TRIzol (Ambion), according to the manufacturer’s protocol, with the exception that all RNA precipitations were carried out at – 80°C for at least 2 h and using 1 µl of glycogen (15 mg/ml; Thermo Fisher Scientific). One µg of total RNA was treated with 10 U of DNase I recombinant (Roche) in a final volume of 12 µl. Samples were incubated at 37°C for 2 h (plasmid DNA transfection) or 45 min (siRNA transfection) followed by 10 min at 75°C for enzyme inactivation. Complementary DNA (cDNA) was synthesized using SuperScript IV Reverse Transcriptase (Thermo Fisher Scientific) and random hexamers (Sigma), according to the manufacturer’s protocol. To rule out genomic DNA contamination, a mixture without reverse transcriptase was also prepared.

Reverse transcription-quantitative polymerase chain reactions (RT-qPCRs) were prepared by combining 1 µl of cDNA with 5 µl of SYBR Select Master Mix (Applied Biosystems), 0.125 µl (0.125 µM) of each primer (Supplementary Table S1), and 3.75 µl of nuclease-free water. Triplicates of each sample, RT^−^ controls, and template negative controls, without cDNA, were amplified on a 7500 Fast Real-Time PCR System (Applied Biosystems), using the 60°C default program recommended by the manufacturer. Real-time PCR primer efficiencies were calculated from the slope obtained by the serial dilutions using the equation: E = 10[–1/slope] [53]. For the primer pairs efficient at 58°C, the program used was as follows: 2 min at 50°C and 2 min at 95°C; 40 cycles at 95°C for 15 s, 30 s at 58°C, and 1 min at 72°C; the melt curve stage occurs at 95°C for 15 s, 1 min at 60°C, 30 s at 95°C, and 15 s at 60°C. The results were analysed using the ΔΔCt method [53] and *18S* was used as housekeeping gene to normalize mRNA levels between different samples.

### Cell culture and transfection

HeLa and HEK293 adherent cell lines were grown and maintained in Dulbecco’s Modified Eagle Medium (DMEM) with GlutaMAX, supplemented with 10% fetal bovine serum (FBS) and 1% penicillin-streptomycin solution (10 000 U/ml) (Gibco, Thermo Fisher Scientific). The cell lines were maintained in a humidified incubator at 37°C with 5% CO_2_, and subcultured every 3 days.

HeLa cells were seeded in 24-well plates and transfected at 70%–90% confluency with 0.5 µg of plasmid DNA (pUC19mini-TOL-ΔDplUSE, pUC19mini-TOL-MCS, pUC19mini-TOL-DplUSE, or pUC19mini-TOL-DplUSEmt) using 1 μl of Lipofectamine™ 2000 Transfection Reagent (Thermo Fisher Scientific), following manufacturer’s instructions. Forty-eight hours post-transfection, the cells were collected for total RNA extraction. HEK293 cells were co-transfected in 24-well plates with 400 ng of reporter plasmid (pLuc-USE non-risk variant or pLuc-USE risk variant) and 100 ng pRL-TK *Renilla* plasmid (Promega), as described earlier. Forty-eight hours post-transfection the cells were harvested for luciferase assays.

To deplete *PTBP1*, two siRNAs (siPTBP1; Sigma–Aldrich; Supplementary Table S1) were transfected into HeLa cells in 24-well plates using 2 μl of jetPRIME^®^ (Polyplus Transfection) reagent, following manufacturer’s guidelines. A non-target siRNA pool (siNTC; Dharmacon; Supplementary Table S1) was also transfected to serve as a negative control. Cells were transfected at 50% confluency with 50 nM of siPTBP1 (25 nM siPTBP1_1 + 25 nM siPTBP1_2) or 50 nM of siNTC and collected for total RNA extraction 48 h post-transfection. To deplete *HuR* and *hnRNP C* from HeLa cells, the correspondent siRNAs (siHuR and sihnRNPC; Ambion; Supplementary Table S1), together with a non-target control (siNTC: Silencer™ Select Negative Control No. 1 siRNA; Ambion), were transfected in 24-well plates using 1 μl of Lipofectamine™ RNAiMAX Transfection Reagent (Thermo Fisher Scientific), following manufacturer’s guidelines. Cells were transfected at 60%–80% confluency with 5 pmol of siHuR, sihnRNP C, or siNTC and collected for total RNA extraction 48 h post-transfection.

### RNA fractionation

Subcellular RNA fractionation was adapted from [54], where HeLa cells from three wells of a 24-well plate were trypsinized, washed two times in ice-cold PBS 1×, and the cell pellet was then resuspended with slow pipetting in 500 µl of Lysis Buffer 1 [10 mM Tris–HCl (pH 8.4), 140 mM NaCl, 1.5 mM MgCl_2_, 0.5% NP-40, 1 mM DTT, 100 U/ml Ribolock]. The nuclear suspension was centrifuged at 1000 × *g* for 4 min at 4°C. The supernatant (cytoplasmic fraction) was removed and centrifuged at 11 000 × *g* for 1 min at 4°C to remove remaining nuclei. TRIzol was added to the cytoplamisc fraction and RNA extraction was performed according to the protocol described earlier. The nuclear fraction was resuspended in 1 ml of Lysis Buffer 2 (900 µl of Lysis Buffer 1 with 100 µl of 3.3% (w/v) sodium deoxycholate and 6.6% (v/v) Tween 20) were added under slow vortexing. The nuclei were centrifuged at 1000 × *g* for 4 min at 4°C. The nuclei pellet was then washed once more in 500 µl of Lysis Buffer 1 and centrifuged at 1000 × *g* for 4 min at 4°C. The nuclei pellet was then resuspended in 500 µl of TRIzol. To verify successful subcellular RNA fractioning, 1 µg of nuclear and cytoplasmic RNA were run on a 1.2% agarose gel, at 100 V for 20 min.

### Luciferase assays

Luciferase assays were performed using the Dual-Luciferase Reporter Assay System (Promega) following the manufacturer’s protocol. First, transfected HEK293 cells were rinsed with PBS 1× and 100 µl of 1× Passive Lysis Buffer was added to each well on a 24-well plate, followed by 15 min of shaking at room temperature. Cell lysates were transferred to microtubes, centrifuged for 5 min at 4°C at 14 000 × *g*, and the supernatant of each sample was collected into a new microtube. Then, 10 µl of each sample was placed on a 96-well plate and Firefly luciferase activity was measured by adding 50 µl of LAR II to each well. Next, 50 µl of Stop & Glo Reagent was added to each sample to perform the *Renilla* luciferase activity measurements. Luminescent measurements of both Firefly and *Renilla* luciferase activities were performed in a Synergy 2 Multi-Mode Reader (Bio-Tek). Firefly luciferase activity was normalized to the *Renilla* luciferase activity.

### Bioinformatics RNA-seq and DGE analysis

For RNA sequencing, total RNA was extracted by TRIzol (Thermo Fisher Scientific), treated with DNase I (Merck) to remove DNA, and purified with acidic phenol/chloroform (Thermo Fisher Scientific). Quantity of the purified RNA was determined by Qubit (Thermo Fisher Scientific) and RNA integrity was assessed by Bioanalyzer 2110 (Agilent) with all samples displaying a RIN > 7. Library preparation and RNA sequencing were performed by Biomarker Technologies (BMK) GmbH. RNA-seq analysis was performed in a total of 10 samples: 5 samples of HeLa cells with PTBP1 depletion (siPTBP1) and 5 samples of HeLa cells transfected with an siRNA control (siNTC). The raw sequencing data were obtained in FASTQ format. Quality assessment of the reads was carried out using FastQC [55]. Adapter trimming and quality filtering were conducted using fastp [56]. The cleaned reads were then aligned to the human reference genome (hg38, UCSC) using the splice-aware HISAT2 [57]. Post-alignment, SAM files were converted to BAM format, sorted, and indexed using SAMtools [58]. Duplicate reads were identified and removed using the GATK toolkit [59].

Gene-level read quantification was performed using HTSeq-count [60], utilizing the corresponding gene annotation file in GTF format from UCSC. The resulting count matrices were used for differential gene expression analysis with DESeq2 [61] in R. Genes with an adjusted *P*-value (FDR) < .05 and a log_2_foldchange >0.5 and <-0.5 were considered differentially expressed.

Additionally, from the DplUSE-containing human genes, the ones that were expressed in HeLa were identified using a threshold of RPKM ≥ 0.5. Then, these expressed genes were intersected with the differentially expressed genes using Venn diagrams created in R with the ggvenn package (https://github.com/NicolasH2/ggvenn).

### CLIP datasets analysis

To assess whether hnRNPC, PTBP1, and HuR bind to the DplUSE sequence, we analysed CLIP datasets generated from HeLa cells in three independent studies (HuR [62], Gene Expression Omnibus accession number GSE29943; hnRNPC [63], ArrayExpress accession number E-MTAB-1371; PTBP1 [64], Gene Expression Omnibus accession number GSE19323). Using the corresponding BED files, we intersected RBP-binding coordinates with the DplUSE sequence, considering a positive hit only when the entire DplUSE sequence was encompassed within the RBP-binding region. The percentage of DplUSE sequences bound by each RBP was then calculated. To test whether RBP binding was enriched at the DplUSE motif, we evaluated binding across the broader 3′UTR context, independently of the DplUSE motif, by generating a 7-nt sliding window spanning the full 3′UTRs of transcripts containing the DplUSE motif. These windows were intersected with the same CLIP datasets to calculate the percentage of windows bound by each RBP. To identify co-binding of RBPs at the same DplUSE sequence, we searched for overlapping RBP-binding coordinates within each DplUSE. To test the flexibility of the DplUSE sequence in binding hnRNPC, PTBP1, and HuR, we applied a strategy similar to that used for identifying DplUSE sequences, maintaining the maximum distance of 450 bp from the PAS, but searching for the DplUSE variants described in Fig. 4M. These coordinates were then intersected with RBP-binding sites from the CLIP datasets, and the percentage of sequences of each variant bound by each RBP was calculated.

### Statistical analysis

Statistical analyses were performed using unpaired Student’s *t*-test and by using χ^2^ test with Fisher correction or one-way ANOVA corrected for multiple comparisons using the Tukey multiple comparisons test. *P*-values < .05 were considered statistically significant: *, *P *< .05; **, *P *< .01; ***, *P *< .001; ****, *P *< .0001.

## Results

### A fruit fly USE is conserved in the 3′UTR of human, mouse, and zebrafish genes

Human USEs have been identified upstream of a range of PASs, and these have an auxiliary function in pre-mRNA 3′ end processing through RBPs (summarized in Table 1). USEs are localized towards the 3′ ends of human genes/transcripts [34, 65], as they enhance the efficiency of weak PASs, for example *COX-2* [23, 30], and promote the usage of proximal PASs in the context of competing PASs [66]. USEs can also exert their function in pre-mRNAs where the PAS is canonical (AAUAAA), as in *C2 complement* [16, 40] and *prothrombin* [33, 34]. Although the canonical AAUAAA is present in these pre-mRNAs, strong U- or G/U-rich DSEs may be absent, and efficient mRNA 3′ end formation therefore depends on auxiliary sequences such as USEs [16, 33, 40].

**Table 1.** Human USEs, their functions, localization and interacting RNA-binding proteins

Gene	Sequence	Role	Localization	Bound RBPs	References
*C2 complement*	5′-GACUUGACUCAUGCUUGUUUCACUUUCACAUGGAAUUUCCCAGUUAUGAAAUU-3′	Enhances cleavage and polyadenylation; required for the full activity of the poly(A) site; required for efficient UV crosslinking of CstF-64 to the PAS.	43 nt upstream of the AAUAAA PAS	PTBP1 (*binds to the underlined sequence*)	(Moreira, Wollerton *et al.* 1995, Moreira, Takagaki *et al.* 1998)
*Lamin B2*	5′-AUUCGGUUUUUAAGAAGAUGCAUGCCUAACGUGUUCUUUUUUUUUUCCAAUGAUUUGUAAUAUACAUUUUAUGACUGGAAACUUUUUUGUACAACACUCC-3′	Required for cleavage and polyadenylation and the production of full-length poly(A) tails; stabilizes the binding of the CPSF to the pre-mRNA.	Immediately 5′ to the AAUAAA PAS	hnRNP CNon-identified 55 kDa protein (*bind to the underlined sequence*)	(Brackenridge and Proudfoot 2000)
*Prothrombin* (F2)	5′-AUUAUUUUUGUGUUU-3′	Stimulates 3′ end processing in a position- and sequence-dependent manner; and a context-independent manner; functionally links the splicing and 3′ end processing machineries.	17 nt from the AAUAAA PAS	U2AF65PTBP1 (*bind to the underlined sequence*)FBP1FBP2FBP3HuRCUGBP-1TIA-1TIAR	(Danckwardt, Gehring *et al.* 2004, Danckwardt, Kaufmann *et al.* 2007, Danckwardt, Gantzert *et al.* 2011)
*COX-2*	5′-AUUUGAA-3′5′-AUUUCUAA-3′5′-AUUUCUUA-3′	Necessary for the processing of the proximal PAS *in vivo*, by recruiting *trans*-acting factors necessary for RNA cleavage and polyadenylation.	The first USE is 57 nt from the proximal AUUAAA PAS	PSFp54^nrb^,PTBP1U1A	(Hall-Pogar, Zhang *et al.* 2005, Hall-Pogar, Liang *et al.* 2007)
*COL1A2*	5′-AUUGUAC-3’5’-AUUUUGUAUA-3’5’-AUUGUGA-3’	Auxiliary polyadenylation efficiency element; helps in the processing of the proximal PAS.	The first USE is 24 nt upstream of the proximal AAUAAA PAS	*Not described yet*	(Natalizio, Muniz *et al.* 2002)
*COL1A1*	5′-AUUUUGUAU-3′5′-AUUUGAGA-3′5′-AUUUUGAUU-3′	*Not defined yet*	The first USE is 43 nt upstream of the AAUAAA PAS	*Not described yet*	
*COL2A1*	5′-AUUCUGU-3′5′-AUUGAU-3′	*Not defined yet*	The first USE is 39 nt from the AUUAAA PAS	*Not described yet*	
*MeCP2*	5′-UUUGUACC-3′	Aids in the processing efficiency of the distal PAS	84 nt upstream of the distal UAUAAA PAS	*Not described yet*	(Newnham, Hall-Pogar *et al.* 2010)

In a previous study, we identified a USE located upstream of the proximal PAS of the cell cycle gene *polo* in *Drosophila melanogaster* [15]. We found that this sequence is essential to achieve Polo protein levels required for cell cycle progression [15], indicating that it regulates gene expression.

We have developed a bioinformatic script to analyse the frequency of DplUSE and its distance within 3000 bp upstream of the PAS (*polo*’s pA1, ATTAAA) in the 3′UTRs of human, zebrafish, and mouse mRNAs, and plotted the frequency of these distances in bins of 50 bp (Supplementary Fig. S1A–C). The regions represented in the graphs correspond to 5329 human genes, 4162 mouse genes, and 2599 zebrafish genes, and the average size of the 3′UTRs included in the bins for distances up to ~600 bp is similar. Our results show DplUSEs are mainly located near PASs, confirming previous results with human mRNAs [13, 34]. Notably, in zebrafish, the enrichment of DplUSEs close to PASs is even more evident, indicating that the genomic architecture of these regulatory sequences is conserved in zebrafish.

We then developed another bioinformatic script to identify all the genes that contain the DplUSE at a maximum distance of 450 nt upstream of the non-canonical PAS ATTAAA (Fig. 1A). We chose this genomic architecture as (i) it is similar to *Drosophila’s polo*, where DplUSE was found [15], (ii) USEs are located near the PAS (Table 1, Supplementary Fig. S1A–C, and [16, 20, 23, 27, 34, 67]); (iii) USEs are auxiliary elements that control non-optimal PAS efficiency in humans and *Drosophila* [15, 23, 30, 67], and therefore the chosen PAS corresponds to the non-canonical proximal PAS in *Drosophila*’s *polo* [15]; and (iv) the median length of the 3′UTR is ~600 nucleotides for single-UTR genes [47]. Our bioinformatic search identified the presence of the DplUSE in 2110 human genes, 1648 mouse genes, and 714 zebrafish genes, with subgroups sharing orthologues between species (Fig. 1B). Of note, the 450 nt distance filter in the script decreases only moderately the number of genes that contain a USE within 450 nt of the PAS to approximately one-third in comparison to the number of genes obtained with the 3000 bp distance (Supplementary Fig. S1A–C). From the 2110 human genes that contain DplUSE, we observed a significant enrichment for genes involved in diseases such as congenital abnormalities, colorectal cancer, and malignant neoplasms (MNeoplasms), compared with the control genes (all genes associated with the respective disease, with or without DplUSE; Fig. 1C). Importantly, 31 orthologue genes in human, mouse, and zebrafish share the presence of the DplUSE in their 3′UTR (Fig. 1B and D) and are also enriched for several diseases, compared with the control genes (Supplementary Fig. S1D). Altogether, these results highlight the potential of the involvement of DplUSE function in human diseases. Considering this, we then explored whether the DplUSE sequence could show a higher level of conservation than the respective 3′UTR region, when aligned with multiple vertebrate genomes. The results showed that indeed the DplUSE motif is significantly more conserved (Fig. 1E and Supplementary Fig. S1E and F), when compared to the remaining 3′UTR, especially in the 31 orthologous gene dataset (Fig. 1E). Within the 31-orthologue dataset, 21 genes (68%) showed higher conservation values for the DplUSE compared to the 3′UTR, while in the remaining 10 genes (32%), the 3′UTR region exhibited higher conservation than the DplUSE region. The observation that the DplUSE is more conserved than the 3′UTR in 31 genes that are common to three highly evolutionarily divergent species such as human, mouse, and zebrafish supports a conserved regulatory function for this non-coding sequence in vertebrates. Next, we evaluated the degree of conservation of each nucleotide of the DplUSE motif in vertebrates based on the multiple alignments of 100 vertebrate species and measurements of evolutionary conservation using phyloP [68]. For this analysis we extended 3 nucleotides (N) upstream and downstream of DplUSE. Using the DplUSE motifs identified in the 2110 human DplUSE-containing genes, we observed that the TTT located in the 5th to 7th positions of the DplUSE motif (TTGTTTT) present highest values of conservation, while the T located in the 4th position of the motif presents higher flexibility, showing the lowest value of conservation (Fig. 1F). Similar results were observed when analysing the DplUSE motif present in the 31 genes subset (Supplementary Fig. S1G). These results suggest that the DplUSE motif might accommodate some nucleotide variants, as shown for other USEs (Table 1). Nevertheless, using the multiple DplUSE human sequences aligned with the genomes of key vertebrate species (mouse, alligator, and zebrafish), we found that the most constant consensus was the TTGTTTT motif, with the exception of the alignments with zebrafish, for which motif was TTCTTTC (Supplementary Fig. S1H and I). Analyses of the gene ontology (GO) terms related to biological processes demonstrated that *Homo sapiens* DplUSE-containing genes are involved in dynamic processes such as cellular migration, adhesion and communication, and mRNA processing (Supplementary Fig. S1J). For *Mus musculus*, DplUSE-containing genes GO terms include regulation of epithelial to mesenchymal transition and cell response to glucose (Supplementary Fig. S1K), and for *Danio rerio*, DplUSE-containing genes are involved in mitotic cell cycle process, protein ubiquitination, and several developmental processes (Supplementary Fig. S1L). We then asked if there were common GO terms that are highly enriched (F.E. ≥ 1.5) between the three organisms (Supplementary Fig. S1M). Although there are no common GO terms for the three species altogether, we found that 10 out of 22 human GO terms are common to mouse, 2 are common to zebrafish GO terms, and 4 out of 14 zebrafish GO terms are common to mouse (Supplementary Fig. S1M and N). These GO terms include cell-cycle-related functions such as ‘cell division’ (human and mouse), ‘chromosome organization’ (mouse and zebrafish), ‘mitotic cell cycle process,’ and ‘mitotic cell cycle’ (human and zebrafish) (Supplementary Fig. S1N), which is consistent with the role of the fruit fly gene *polo*, in which the DplUSE was originally identified, suggesting that DplUSE might have conserved regulatory functions in cell cycle control. GO terms for other important cell biology terms such as ‘positive regulation of cell migration’ and ‘mRNA processing’ have also been identified (Supplementary Fig. S1N).

### The DplUSE sequence increases gene expression in a vertebrate animal model

To understand if the DplUSE might increase gene expression in vertebrates as observed in the fruit fly, we constructed two expression vectors, pUC19miniTOL-GFP-DplUSE and pUC19miniTOL-GFP-DplUSEmt. These constructs are ubiquitous expression vectors containing the DplUSE sequence or the DplUSE sequence mutated in 17 nucleotides (DplUSEmt), subcloned downstream of the *GFP* coding sequence and assembled in a Tol2 transposon (Fig. 1G), suitable to perform transgenesis reporter assays in zebrafish embryos. DplUSEmt corresponds to the mutations introduced in our previous study in *Drosophila* [15], where we showed that it affects the expression of *polo, in vivo*. In DplUSEmt, the pyrimidines were mutated to purines, as in humans USEs are pyrimidine-rich (Table 1) [16, 20, 23, 27, 34, 67].

Microinjection of these constructs into one-cell stage wild-type (WT) embryos, simultaneously with *Tol2* mRNA and another transposon that drives expression of mCherry in the muscle (control of transgenesis), showed that at 24 h post-fertilization (24 hpf), embryos microinjected with GFP-DplUSE DNA exhibited more intense GFP fluorescence, while embryos microinjected with GFP-DplUSEmt DNA demonstrated a more diffused and faded fluorescence (Fig. 1H). Quantification of GFP intensity (Fig. 1J, left) revealed that embryos microinjected with GFP-DplUSE DNA showed a significantly higher expression than when microinjected with GFP-DplUSEmt DNA. These results demonstrate that DplUSE enhances *GFP* expression in a vertebrate species as in the fruit fly, demonstrating conserved function as a positive regulator of gene expression, from arthropods to vertebrates.

To better understand if this activating effect functions at a transcriptional or post-transcriptional level, *in vitro* transcribed *GFP-DplUSE* and *GFP-DplUSEmt* RNAs were microinjected into one-cell stage zebrafish embryos (Fig. 1I). Although fluorescence in these embryos was more diffuse than in embryos microinjected with expression vectors (Fig. 1H), it was possible to detect higher GFP expression in embryos microinjected with *GFP-DplUSE* RNA than with *GFP-DplUSEmt* RNA (Fig. 1I and J, right). These results indicate that, in zebrafish, the DplUSE sequence increases gene expression in a post-transcriptional manner.

### *DplUSE* RNA recruits protein factors essential for the correct expression of genes involved in zebrafish development

To better characterize the function of the DplUSE sequence in controlling gene expression, a transgenic GFP-DplUSE zebrafish line was generated using the Tol2 transposon system [69]. Although the expression of GFP is driven by a ubiquitous promoter (CMV), the stable transgenic line exhibits GFP expression mainly in the notochord, muscle, pituitary gland, and eye (Fig. 2A and B), suggesting a possible role of the DplUSE in regulation of gene expression in a tissue- and cell-type-specific manner. Specifically, confocal imaging demonstrates a clear and intense pattern of GFP expression in the notochord and muscle (Fig. 2B). To address the function of the DplUSE and the proteins that recognize this sequence, we considered a dominant negative approach, induced by ectopic introduction of *DplUSE* RNA. In this case, we postulated that factors such as RBPs might be sequestered by the ectopic *DplUSE* RNA, thereby affecting the expression of DplUSE-controlled genes. To test this hypothesis, we microinjected *DplUSE* RNA in transgenic GFP-DplUSE embryos and quantified *GFP* expression, in comparison with microinjection of *DplUSEmt* RNA (DplUSE sequence mutated as in Fig. 1G) and non-injected embryos. We found that the microinjection of *DplUSE* RNA was able to decrease GFP expression, contrasting with non-microinjected embryos or the microinjection of *DplUSEmt* RNA at 5 dpf, particularly in the notochord and in the muscle (Fig. 2C–E), therefore corroborating the dominant negative effect hypothesis. These results were consistent at 48 hpf, where a decrease in GFP expression in the notochord was also detected (Supplementary Fig. S2A and B). Microinjection of *DplUSE* RNA also affected the consistency of the GFP expression, leading to the development of patchy GFP expression, a phenomenon that occurred less frequently upon microinjection of *DplUSEmt* RNA at 5 dpf (Fig. 2C and F) and at 48 hpf (Supplementary Fig. S2A and C), and in non-microinjected embryos (Fig. 2F and Supplementary Fig. S2C).

**Figure 3.**
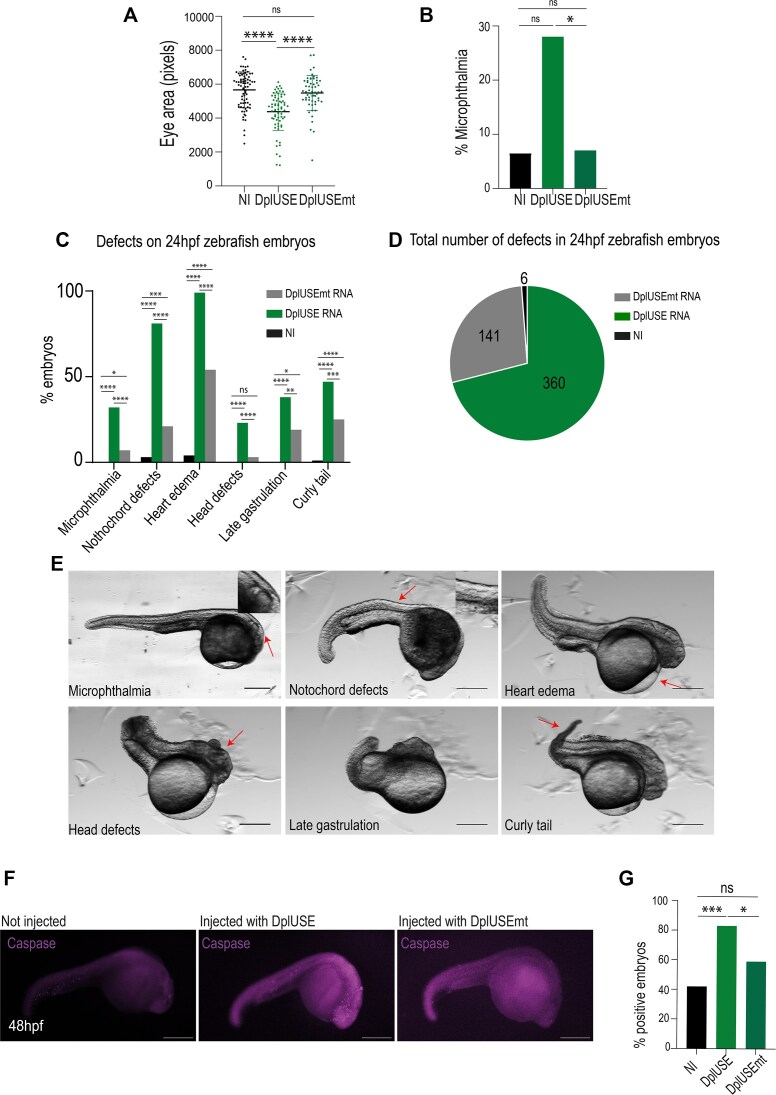
Microinjection of *DplUSE* RNA in one-cell-stage zebrafish embryos induces the development of several defects. (**A**) Representative graph of the quantification of eye size (pixels) in non-microinjected and microinjected embryos with *DplUSE* and *DplUSEmt* RNAs at 5 dpf (*n* = 78 Non-Injected (NI); *n* = 69 DplUSE; *n* = 58 DplUSEmt). (**B**) Representative graph of the quantification of the percentage of microphthalmia in non-microinjected and microinjected embryos with *DplUSE* and *DplUSEmt* RNAs at 5 dpf (6.5%, *n* = 79 NI; 28%, *n* = 68 DplUSE; 7%, *n* = 58 DplUSEmt). (**C**) Quantification of the several defects developed in non-microinjected (in black) one-cell stage zebrafish embryos and 24 h upon the microinjection of *GFP-DplUSE* RNA (in green) and *GFP-DplUSEmt* RNA (in grey). (**D**) Pie chart representing the total number of defects developed without microinjection and upon the microinjection of *GFP-DplUSE* RNA and *GFP-DplUSEmt* RNA. (**E**) Representative images showing the various abnormalities upon microinjection of *GFP-DplUSE* RNA at 24 hpf, indicated with red arrows. (**F**) Representative images of not injected embryos and embryos injected with *DplUSE* and *DplUSE* RNAs, at 48 hpf, showing in purple, the cleaved anti-caspase-3 staining. (**G**) Representative graph showing the percentage of embryos with consistent appearance of apoptotic cells, characterized by the presence of anti-caspase-3 staining (42%, *n* = 39 not injected; 83%, *n* = 33 DplUSE; 59%, *n* = 33 DplUSEmt). Statistical significance was determined by χ^2^ test with Fisher correction or by two-tailed unpaired t-test. ***P < .001; **P < .01; *P < .05; ns *P *> .05 Images acquired with Leica M205. Scale bar = 100 μm.

Next, we microinjected *DplUSE* RNA into WT zebrafish embryos and analysed the effect on the phenotypes developed by the embryos. At 5 dpf, zebrafish larvae microinjected with *DplUSE* RNA exhibited microphthalmia, a condition where either one or both eyes are abnormally small or absent [70]. When the eye size was quantified, a significant decrease was observed compared to larvae microinjected with *DplUSEmt* RNA and non-microinjected larvae (Fig. 3A and B). This phenotype was also observed at 48 hpf (Supplementary Fig. S2D and E). Apart from these eye phenotypes, a careful evaluation of 24 hpf larvae allowed us to identify other important phenotypes affecting the notochord, heart, head, late gastrulation, and tail that are more prevalent in *DplUSE* RNA than with *DplUSEmt* RNA-injected embryos and non-injected embryos (Fig. 3C–E). At 5 dpf, malformations in the notochord, head, tail, and swim bladder were also detected (Supplementary Fig. S3A–C). To better understand the mechanisms that might be causing these phenotypes, we performed immunohistochemistry staining with anti-cleaved caspase-3 polyclonal antibody (pAb) and analysed the presence of cleaved caspase-3 fluorescence signal in 48 hpf embryos microinjected with *GFP-DplUSE* and *GFP-DplUSEmt* RNAs to analyse apoptosis. We observed more cleaved caspase-3 staining in embryos microinjected with *GFP-DplUSE* RNA, compared to not-injected embryos and embryos injected with *GFP-DplUSEmt* RNA (Fig. 3F–G and Supplementary Fig. S3D), suggesting that, in part, these phenotypes are caused by apoptosis. Overall, these results suggest that ectopic *DplUSE* RNA works as a dominant negative, interfering with the normal DplUSE regulatory functions, as shown using the GFP-DplUSE reporter line (Fig. 2). In addition, ectopic *DplUSE* RNA causes several developmental abnormalities, possibly by sequestering RBPs that bind to the *DplUSE* mRNA of endogenous DplUSE-containing genes, interfering with the expression of the genes necessary for correct embryo development.

### DplUSE activates gene expression in HeLa cells and binds HuR, hnRNP C, and PTBP1 *in vitro*

**Figure 4.**
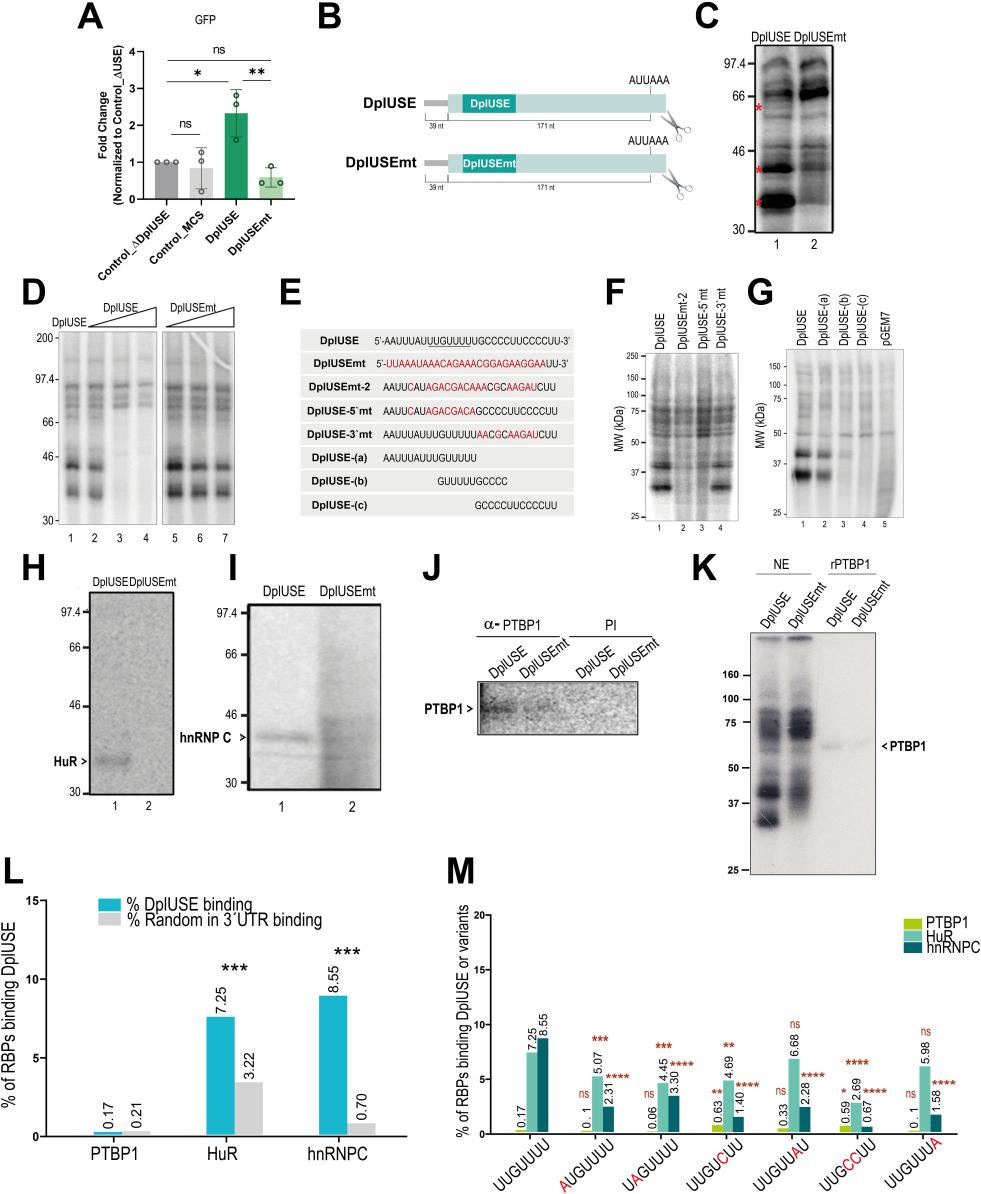
DplUSE increases gene expression in human cells by binding to RBPs. (**A**) RT-qPCR showing that the mRNA levels of GFP increase by over two-fold upon transfection with the pUC19miniTOL-GFP-DplUSE plasmid (DplUSE) when comparing with the transfection with the pUC19miniTOL-GFP-ΔDplUSE (Control_ΔDplUSE) plasmid, the pUC19miniTOL-GFP-MCS (Control_MCS) or the pUC19miniTOL-GFP-DplUSEmt (USEmt) plasmid. The data are presented as the mean ± SD of three independent experiments (*n* = 3) and statistical significance was determined by a one-way ANOVA corrected for multiple comparisons using the Tukey multiple comparisons test. (**B**) Schematic representation of the pre-cleaved pre-mRNAs used to perform the UV crosslinking assays with HeLa cell nuclear extracts. The DplUSE sequence and the DplUSE sequence mutated in 26 nucleotides (DplUSEmt) are presented. The cleaved pre-mRNAs were obtained by linearizing the plasmids with restriction enzymes in proximity to the cleavage site (portrayed by the pair of scissors). The conserved heptamer of the DplUSE sequence (UUGUUUU) is underlined. (**C**) UV crosslinking assay using the pre-cleaved pre-mRNAs *DplUSE* and *DplUSEmt*. Asterisks define the bands corresponding to the proteins that undergo a decrease in the binding upon the mutation of the DplUSE sequence to DplUSEmt. (**D**) UV crosslinking competition assay using pre-cleaved pre-mRNAs *DplUSE* and *DplUSEmt* (lane 1) and an increasing amount of unlabelled *DplUSE* (lanes 2–4) and *DplUSEmt* (lanes 5–7) pre-mRNAs. One (lanes 2, 5), 50 (lanes 3, 6), and 150 pmols (lanes 4, 7) of unlabelled oligonucleotides were used to compete with the labelled pre-mRNAs. (**E**) DplUSE, with the most conserved sequence (UUGUUUU) underlined, and the mutated DplUSE sequences used for UV-crosslinking analysis. (**F**) UV crosslinking assays using *DplUSE* (lane 1), *DplUSEmt-2* (lane 2), *DplUSE5’-mt* (lane 3), and *DplUSE3’mt* (lane 4) pre-cleaved pre-mRNAs. (**G**) UV crosslinking assays using *DplUSE* (lane 1), *DplUSE-(a)* (lane 2), *DplUSE-(b)* (lane 3), and *DplUSE-(c)* (lane 4) pre-cleaved pre-mRNAs and the control *pGem7* RNA (lane 5). (**H**–**J**) Immunoprecipitation assays performed subsequently to UV crosslinking of pre-cleaved pre-mRNAs *DplUSE* and *DplUSEmt* with HeLa cell nuclear extracts. Immunoprecipitations were performed using HuR (**H**) and hnRNP C (**I**) monoclonal and PTBP1 (**J**) polyclonal specific antibodies. As a control for the immunoprecipitation with the PTBP1 polyclonal antibody, the pre-immune (PI) serum was used. (**K**) UV crosslinking assays were performed using HeLa nuclear extracts (NE) and 50 ng of recombinant human PTBP1 (kind gift of Chris Smith). (**L**) Percentage of DplUSE sequences and 100 000 random 7-bp sequences in the 3′UTRs of 4634 DplUSE-containing transcripts bound by HuR [62], hnRNPC [63], or PTBP1 [64], as detected by CLIP in HeLa cells. Statistical significance of the annotated comparisons was assessed with a Fisher’s exact test. (**M**) Percentage of DplUSE sequences and their variants (nucleotide changes highlighted in red) bound by HuR, hnRNPC, or PTBP1, as detected by CLIP in HeLa cells. Statistical significance was assessed with a Fisher’s exact test relative to the DplUSE sequence. Statistical differences are considered when *P *< .05, and in *P < .05, **P < .01, ***P < .001, ****P < .0001 and ns: not significant.

To understand if the DplUSE sequence has a similar role in human cells as observed in the fruit fly and zebrafish, we started by asking whether the DplUSE would activate *GFP* expression in HeLa cells, using reporter constructs. Although the DplUSEmt is an effective control for the DplUSE [15], we also evaluated two other controls: Control_ΔDplUSE is the DplUSE plasmid with a deletion of the DplUSE motif, and Control_MCS is the DplUSE plasmid, where the DplUSE sequence was substituted by a DNA fragment of a similar length isolated from a vector polylinker, to rule out possible spacing effects. Transfection with the pUC19miniTOL-GFP-DplUSE and pUC19miniTOL-GFP-DplUSEmt constructs shows that, similarly to what we observed in zebrafish, the DplUSE sequence enhances the expression of *GFP* by over 2-fold in comparison to the controls (Control_ΔDplUSE, Control_MCS) and to DplUSEmt (Fig. 4A). All the controls (Control_ΔDplUSE, Control_MCS, and DplUSEmt) behave similarly (Fig. 4A). Moreover, the lack of statistical significance between all the controls, including DplUSEmt, indicates that the increase in *GFP* expression observed is specifically due to the DplUSE sequence. These results recapitulate our observations in zebrafish, by showing that DplUSE activates *GFP* expression in a human cell line.

Next, we aimed to better understand the molecular mechanism of the DplUSE sequence in controlling gene expression. We reasoned that, because of its nature as a USE located at the 3′UTR of genes, the DplUSE could be binding RBPs that have been shown to be players in USE function [23, 27, 34, 40, 71]. To determine if human RBPs bind to the DplUSE, constructs containing the DplUSE or DplUSEmt sequences were used to perform *in vitro* transcription, generating radioactively labelled pre-mRNAs that were incubated with human HeLa cell nuclear extracts to perform UV crosslinking assays (Fig. 4B). The results show that multiple proteins recognize the *DplUSE* pre-mRNA (Fig. 4C, lane 1) and that mutation of the DplUSE sequence leads to reduced intensity of some of the protein bands (Fig. 4C, lane 2). To determine the binding specificity of these proteins to the *DplUSE* RNA motif, UV crosslinking competition assays were performed, where increasing amounts of unlabelled *DplUSE* or *DplUSEmt* RNA were added to the UV crosslinking reaction mix (Fig. 4D). The results showed that *DplUSE* RNA unlabelled competitor (1 pmol, 50 pmol, and 150 pmol—Fig. 4D, lanes 2, 3, and 4, respectively) significantly inhibits the assembly of the ∼35 kDa, ∼40 kDa, and ∼60 kDa proteins onto the radioactive labelled *DplUSE* pre-mRNA. This competition is DplUSE sequence specific, as addition of the same amounts of unlabelled *DplUSEmt* RNA (1 pmol, 50 pmol, and 150 pmol—Fig. 4D, lanes 5, 6, and 7, respectively) did not cause any noticeable changes in the proteins bound to *DplUSE* pre-mRNA. To map the binding sites of the RBPs within the DplUSE sequence, we introduced specific mutations at the 5′ half of the sequence (DplUSE-5′mt), at the 3′ half (DplUSE-3′mt) and at the both 5′ and 3′ halves together (DplUSEmt-2) (Fig. 4E), and performed UV crosslinking assays with the RNAs. Our results show that the ∼35 kDa, ∼40 kDa, and ∼60 kDa proteins strongly bind to *DplUSE* and *DplUSE-3′mt* RNA sequences, indicating that they bind to the highly conserved UUGUUUU (Fig. 4F). To more precisely map the binding sites of these RBPs, we performed similar assays splitting the expanded DplUSE sequence into three different RNA fragments: *DplUSE-(a), DplUSE-(b)*, and *DplUSE-(c)* (Fig. 4E and G). Results show that the *DplUSE-(a)* containing the UUGUUUU element is sufficient for the binding of most RBPs, in particular the proteins of ∼35 kDa and ∼40 kDa. (Fig. 4G lanes 1–4; RNA transcribed from the pGEM7 polylinker in lane 5 was used as a non-specific sequence control). When we compare *DplUSE-(a), DlpUSE-(b)*, and *DplUSE-(c)* (lanes 2–4), the GUUUUU element seems sufficient for the binding of the ∼40 kDa protein. However, as the binding of this protein is stronger for the *DplUSE-(a)* RNA than for the *DplUSE-(b)* RNA (lanes 2 and 3), these results cannot exclude the possibility that this protein’s optimal binding may require flanking sequences or that the binding of both ∼35 kDa and ∼40 kDa proteins might be cooperative. While it is possible that the mutations in *DplUSEmt* RNA introduced binding sites for other RBPs, we are unable to detect new protein bands binding to *DplUSEmt* RNA in comparison to *DplUSE* RNA. We observe an increase in the intensity of a protein band of ∼66 kDa binding to *DplUSEmt* RNA (Fig. 4C) that is outcompeted by unlabelled *DplUSE* (Fig. 4D). However, this protein seems to be non-specific as it binds to all the other six RNAs used (Fig. 4F and G). Therefore, we focused on RBPs that bind specifically to *DplUSE* and not to *DplUSEmt* <?brk ?> RNA.

To identify the RBPs that bind to the *DplUSE* RNA, we analysed in detail the molecular weights of the proteins assembled onto the *DplUSE* pre-mRNA (Fig. 4C, lane 1 and Fig. 4D). We hypothesized that HuR/ELAVL1 and hnRNP C could be binding to DplUSE based on their characteristics. HuR is a 36 kDa RBP, ubiquitously expressed in all human tissues, that recognizes and binds to U-rich and AU-rich elements present in the 3′UTR of target mRNAs, modulating their stability [35, 72–74]. Furthermore, its fruit fly orthologue Elav has been shown to promote 3′UTR lengthening observed in the nervous system [75]. In addition, hnRNP C1/C2 isoforms (41 and 43 kDa, respectively) bind to polyU tracts and assemble onto nascent transcripts and modulate several mRNA functions, including mRNA stability [36, 76, 77]. Using antibodies against these proteins it was possible to immunoprecipitate these RBPs after UV crosslinking using the *DplUSE* pre-mRNA and HeLa cell nuclear extracts (Fig. 4H and I). In contrast, when the UV crosslinking assays were performed with *DplUSEmt* pre-mRNA, HuR does not bind, and hnRNP C binds more weakly, as evidenced by the lower intensity of the immunoprecipitated bands (Fig. 4H and I). In the fruit fly, the RBP Heph was previously shown by us to bind to *DplUSE* RNA (and not to the mutant version of this element, *DplUSEmt*) from the fly gene *polo* [15] and is required for DplUSE function *in vivo* [15]. For this reason, we tested whether the human orthologue of Heph, PTBP1, could bind to the *DplUSE* RNA *in vitro* using HeLa cell extracts. Immunoprecipitation assays after UV crosslinking show that similarly to Heph, the human orthologue PTBP1 binds more strongly to the *DplUSE* than to the *DplUSEmt* control RNA (Fig. 4J). To confirm this result, we performed UV-crosslinking assays using recombinant hPTBP1 (Fig. 4K, lanes 3 and 4). Our results show that recombinant hPTBP1 binds to *DplUSE* RNA and that the mutation in DplUSE reduces its binding, revealing its specificity. Overall, these results show that human HuR, hnRNPC and PTBP1 bind to *DplUSE* RNA *in vitro*, and are likely to be involved in the function of DplUSE.

### Human HuR and hnRNP C bind to *DplUSE* RNA *in vivo*

To gain a more comprehensive understanding of DplUSE function *in vivo*, we analysed crosslinking immunoprecipitation followed by high-throughput sequencing (CLIP-seq) data from HeLa cells for HuR (iCLIP) [62], hnRNPC (iCLIP) [63], and PTBP1 (CLIP-seq) [64]. We identified 29 438 HuR binding sites, 438 360 hnRNPC binding sites, and 51 388 PTBP1 binding sites. Then, we asked how frequently HuR, hnRNPC, and PTBP1 are detected in DplUSE sequences in 4634 transcripts corresponding to the 2110 human DplUSE-containing genes. We found that HuR (HuR: 7.25%, *n* = 336 out of 4634) and hnRNPC (hnRNPC: 8.55%, *n* = 396 out of 4634) are more frequently detected in DplUSE than PTBP1, which shows binding to a very residual number of DplUSE sequences (PTBP1: 0.17%, *n* = 8 out of 4634) (Fig. 4L and Supplementary Table S2). Additionally, as a control, we analysed the binding of the RBPs to 100 000 randomly selected locations with a length of 7 bp in the 3′UTR of DplUSE-containing transcripts. The results revealed that HuR and hnRNPC bind more frequently and are highly enriched at DplUSEs, while PTBP1 binds less frequently (Fig. 4L). This result for PTBP1 is not unexpected, as it has been shown that PTBP1 binds to 15–25 base stretches rich in Cs and Us [78], and we also showed in our previous work with *polo* that Heph/PTBP1 acts through a 28-nucleotide sequence containing the DplUSE, which is flanked by stretches of cytosines [15].

Next, we analysed the combined binding of HuR, hnRNPC, and PTBP1 at the DplUSE sequence by analysing the CLIP data. Pairwise binding of these RBPs is very rare (HuR–PTBP1: 0.086%, *n* = 4; hnRNPC–PTBP1: 0.065%, *n* = 3; HuR–hnRNPC: 1.575%, *n* = 73 out of 4634; values indicate the percentage of DplUSE sequences with pairwise binding). These findings indicate that the three RBPs are unlikely to be all bound to the same DplUSE sequence within a transcript in a biological context.

We then investigated how flexible the *DplUSE* RNA sequence is in recruiting the same RBPs, using the CLIP data. We quantified the proportion of RBPs that recognize the canonical DplUSE motif and compared it with their binding to 7-nucleotide variants in the 3′UTR differing from the canonical sequence by one or two nucleotides. We found that HuR and hnRNPC binding is more prevalent in the precise sequence of the DplUSE (UUGUUUU) than in similar sequences that diverge in 1 or 2 nucleotides, located in the 3′UTR of any transcript (Fig. 4M).

These results suggest that, *in vivo*, HuR and hnRNPC binding is enriched in the DplUSE sequence in comparison to other regions of the 3′UTRs, while PTBP1 binding is less enriched, thus supporting our *in vitro* results.

### HuR, hnRNP C, and PTBP1 are part of the molecular mechanism modulating the expression of DplUSE-containing genes

To investigate if HuR, hnRNP C, and PTBP1 are required for the control of the expression of DplUSE-containing genes, we depleted the expression of these RBPs in HeLa cells, using siRNAs. A depletion of 96% for hnRNP C, 89% for HuR, and 77% for PTBP1 (Supplementary Fig. S4A) was achieved. We selected six DplUSE-containing genes (*KIF20A, EXT2, CLDN12, MBNL2, MED18, DKC1*) from the 31 orthologous genes identified by the bioinformatic script that are common to human, mouse, and zebrafish (listed in Fig. 1D) and that have a relevant physiological function in malignant neoplasms (Fig. 1C). *KIF20A* is a tumour-associated antigen involved in glioma cell growth and survival [79], *EXT2* has been established as a prognostic and predictive biomarker for head-neck squamous cell carcinoma [80], *CLDN12* is overexpressed in colorectal carcinomas [81], *MBNL2* suppresses tumorigenesis [82], *MED18* is part of the Mediator Complex and is a gastric cancer tumour suppressor [83, 84], and *DKC1* promotes cell proliferation and metastasis formation in colorectal cancer [85, 86].

**Figure 5.**
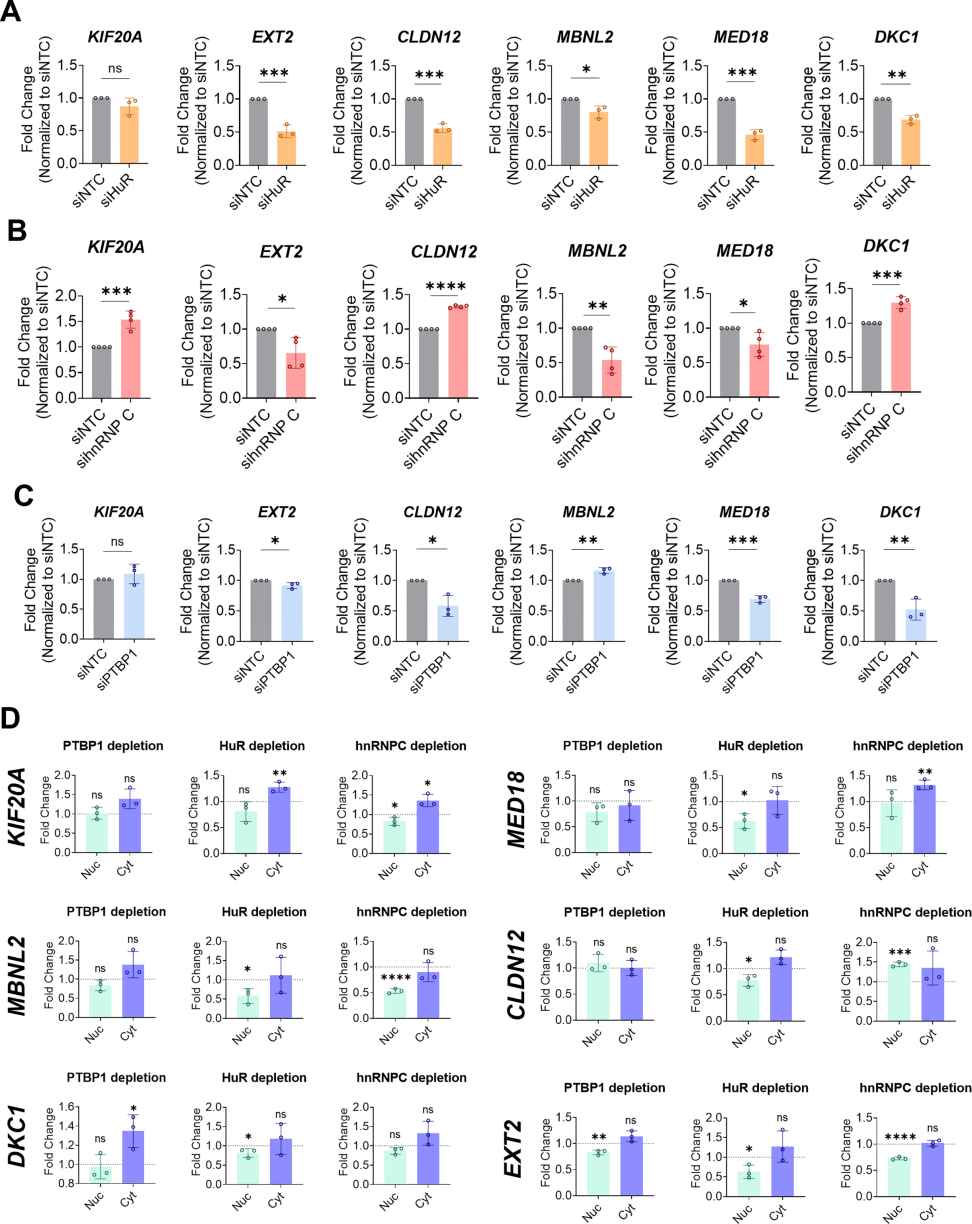
HuR, hnRNP C, and PTBP1 siRNA-mediated depletion modulate the expression of human DplUSE-containing genes. (**A**) HuR, (**B**) hnRNP C, and (**C**) PTBP1 depletion were performed using siRNAs targeting *HuR, hnRNPC*, or *PTBP1* mRNA. As a control, a siNTC was also transfected. The mRNA levels of six DplUSE-containing genes (*EXT2, CLDN12, MBNL2, MED18, DKC1*) were assessed by RT-qPCR for three independent experiments. The mRNA levels were normalized to the internal control *18S*, and fold change was then calculated in relation to the siNTC control. (**D**) DplUSE-containing genes expression analysed by RT-qPCR in HeLa nuclear and cytoplasmic RNA fractions upon PTBP1, HuR, or hnRNPC depletion. The mRNA levels were normalized to the internal control *18S*, and fold change was then calculated in relation to the siNTC control (set as one, represented by the dashed line). The data are presented as the mean ± SD of three independent experiments, analysed by a two-tailed unpaired Student’s t-test. Statistical differences are considered when *P* < .05 and *P < .05, **P < .01, ***P < .001, ****P < .001, and ns: not significant.

HuR siRNA-mediated depletion results in a notable reduction in the mRNA levels from all the genes analysed, with the exception of *KIF20A*, where no significant change is observed (Fig. 5A). These results indicate that in HeLa cells, HuR activates the expression of these DplUSE-containing genes. Regarding hnRNP C depletion, we observe an increase in mRNA levels for three of the DplUSE-containing genes (*KIF20A, CLDN12*, and *DKC1*) and a reduction for the other three genes (*EXT2, MBNL2*, and *MED18*) (Fig. 5B), indicating that hnRNP C is also a modulator of expression of DplUSE-containing genes. Upon siRNA-mediated depletion of PTBP1, we observed that *EXT2, CLDN12, MED18*, and *DKC1* mRNA levels decreased significantly, while *KIF20A* was not clearly affected and *MBNL2* mRNA levels increased (Fig. 5C).

To try to further elucidate the molecular mechanisms involved in DplUSE-mediated gene expression by HuR, hnRNPC, and PTBP1, we applied a rigorous subcellular fractionation strategy (Supplementary Fig. S4B) previously established [54] to analyse differential gene expression in nuclear and cytoplasmic fractions from HeLa cells (Fig. 5D). We used siRNAs for HuR, hnRNPC, and PTBP1 depletion, extracted total RNA from the nuclear and cytoplasmic fractions, and analysed differential expression of the six genes from Fig. 5 A–C. In the nucleus, PTBP1 depletion has little impact on the expression of the six genes, except for *EXT2*, which is downregulated (Fig. 5D). By contrast, HuR depletion reduces the expression of all genes, while hnRNPC depletion decreases the expression of *KIF20A, MBNL2*, and *EXT2*, but increases *CLDN12*. Interestingly, *MBNL2* expression is reduced by ∼50%, indicating that hnRNPC plays a role in regulating *MBNL2* expression. In the cytoplasm, PTBP1 depletion increases *DKC1* without major effects on the other transcripts. HuR depletion does not broadly alter gene expression but increases *KIF20A* levels by ∼25%, while hnRNPC depletion upregulates *KIF20A* and *MED18* (Fig. 5D). Taken together, our results suggest that HuR, hnRNPC, and PTBP1 have gene- and subcellular-specific effects in modulating DplUSE-containing gene expression.

### PTBP1 and hnRNPC modulate alternative polyadenylation of DplUSE-containing genes

**Figure 6.**
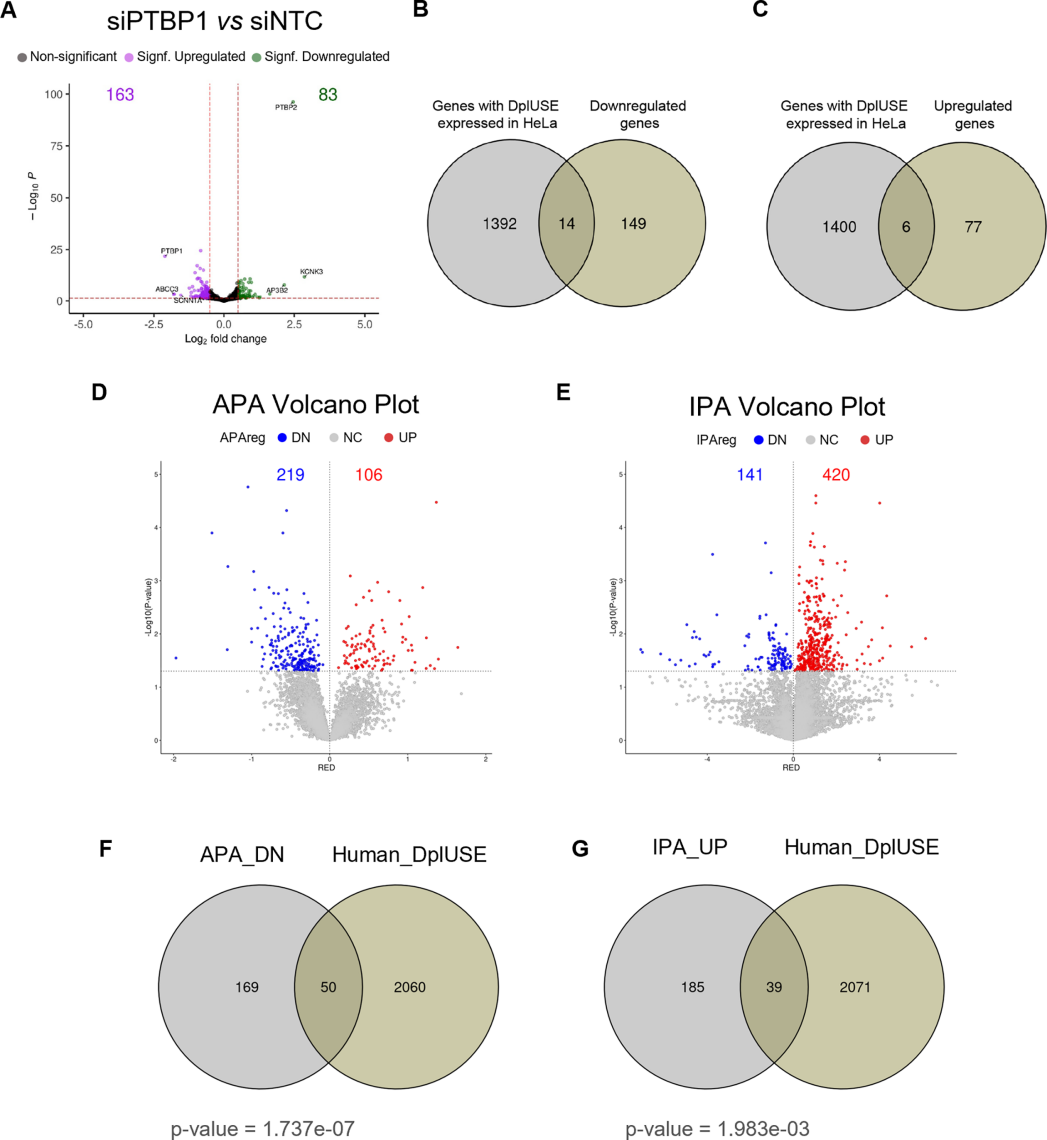
PTBP1 modulares APA and IPA in a DplUSE-dependent manner. (**A**) Volcano plot of differential gene expression (DGE) of PTBP1 depletion (siPTBP1) in comparison to an siRNA non-template control (siNTC) in HeLa cells (*n* = 5). Purple dots represent statistically significant downregulated genes [log_2_Fold Change < -0.5; p adjusted (padj) value < .05], and green dots represent statistically significant upregulated genes (log_2_Fold Change > 0.5; padj < .05). Black dots represent non-statistically significant genes. Venn diagram intersections of the human DplUSE-containing genes only expressed in HeLa cells (1406 genes) with the downregulated (**B**) or upregulated (**C**) genes upon PTBP1 depletion.(**D, E**) Volcano plot of alternative polyadenylation (APA) of PTBP1 depletion (siPTBP1) in comparison to an siRNA non-template control (siNTC) in HeLa cells (*n* = 5). Red dots represent statistically significant genes undergoing lengthening (D) or IPA upregulated genes (E) [RED score > 0; p adjusted (padj) value < .05], and blue dots represent statistically significant shortened genes (D) or IPA downregulated genes (E) (RED score < 0; padj < .05). Grey dots represent non-statistically significant genes. Venn diagram intersections of the human DplUSE-containing genes with the APA downregulated (**F**) or IPA upregulated (**G**) genes upon PTBP1 depletion.

As PTBP1 had been previously described as a USE interactor in humans (Table 1) and with a role in alternative polyadenylation (APA) [15, 40, 87], we further investigated its global function in gene expression and APA by performing RNA-seq on PTBP1-depleted cells. We observed a total of 246 differentially expressed genes, including 163 downregulated and 83 upregulated genes (Fig. 6A). Intriguingly, the DplUSE-containing genes common to human, mouse, and zebrafish are not included in the cohort of genes differentially expressed after PTBP1 depletion. We observed that there are more DplUSE-containing transcripts downregulated than upregulated upon PTBP1 depletion suggesting that PTBP1 acts as a positive regulator of gene expression (Fig. 6B and C and Supplementary Fig. S5A and B).

As PTBP1 has been shown to be involved in 3′ end formation (Table 1), we investigated a possible global function for PTBP1 in APA in HeLa cells. For that, we used APAtizer [88], a bioinformatic tool that enables APA and intronic polyadenylation (IPA) analyses from RNA-seq data. Results show that PTBP1 depletion leads to an increase in the number of genes that preferentially utilize proximal PAS, and also increases IPA, in comparison to non-depleted HeLa cells (Fig. 6D and E and Supplementary Fig. S5C and D). These results indicate that PTBP1 has a global effect on APA and IPA in human cells. Then we asked if the APA and IPA events resultant from PTBP1 depletion occur more frequently in DplUSE-containing genes. We intersected the dataset of DplUSE-containing genes with the dataset of genes undergoing proximal polyA selection and IPA upon PTBP1 depletion. We found that 50 out of 219 genes (23%; *P *= 1.7 × 10^−7^) that select proximal polyA sites and 39 out of 224 genes (17%; *P *= 1.98 × 10^−3^) that undergo IPA contain DplUSE (Fig. 6F and G). These results indicate that the role of PTBP1 in APA and IPA is enriched in DplUSE-containing genes. Taking into account that the CLIP data for PTBP1 shows that it binds very scarcely to the 7-nucleotide *DplUSE* RNA sequence (Fig. 4L), we can assume that, globally, the PTBP1 effect in DplUSE-containing genes’ expression, APA, and IPA is indirect.

As hnRNP C is also a DplUSE interactor (Fig. 4I), we analysed publicly available iCLIP and RNA-Seq from hnRNP C-depleted HeLa cell datasets [63] to investigate a possible global function for hnRNP C in the expression of DplUSE-containing genes. We observed a statistically significant proportion of differentially expressed genes directly bound by hnRNPC within the DplUSE sequence, with a clear enrichment for upregulated genes: 18.5%, i.e. 62 out of 336 DplUSE-containing genes bound to hnRNPC are upregulated (Supplementary Fig. S6A), *versus* 4.5%, i.e. 15 out of 336 DplUSE-containing genes bound to hnRNPC are downregulated upon hnRNPC depletion (Supplementary Fig. S6B). This indicates that hnRNPC modulates the expression of DplUSE-containing genes. As hnRNPC has a function in APA [89, 90], we then used APAtizer in the RNA-seq data and intersected that dataset with the iCLIP dataset. We found that 16% i.e. 54 out of 336 DplUSE-containing genes that bind hnRNP C show differences in APA events in comparison with the siRNA control (Supplementary Fig. S6C). These results indicate that globally hnRNPC has a direct effect not only on DplUSE-containing genes’ expression but also on APA.

Taken together, these results indicate that HuR, hnRNPC, and PTBP1 are all part of a regulatory mechanism that operates at the DplUSE and is conserved in vertebrates.

### A single nucleotide polymorphism in the DplUSE sequence is associated with increased cancer risk

**Figure 7.**
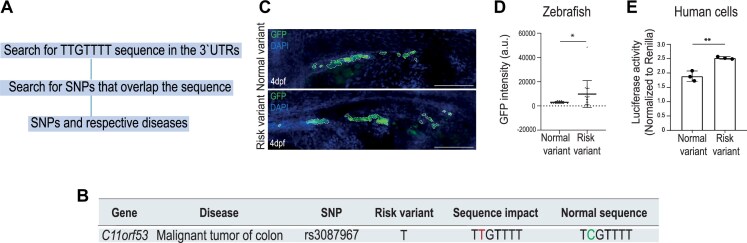
An SNP in *C11orf53* generates an ectopic consensus of the DplUSE, which is associated with malignant tumour of colon. (**A**) Workflow used to identify SNPs that had an associated disease and that overlap with the DplUSE sequence in the 3′UTR. (**B**) DplUSE-related SNP with the respective gene, disease, sequence impact, and normal sequence. (**C**) Representative confocal images of microinjected embryos with GFP-DplUSE of C11orf53 with and without the presence of the risk variant, showing GFP expression in gut cells of 48 hpf zebrafish embryos. The dashed lines represent different GFP expression domains within an embryo. (**D**) Representative graph of the quantification of the GFP expression in the defined GFP expression domains (*n* = 14, normal variant; *n* = 20, risk variant). The images were acquired using the confocal microscope Leica SP5II, and the GFP expression was quantified using IMARIS software. (**E**) Representative graph of Firefly luciferase activity in transfected HEK293 cells (non-risk variant and risk variant DplUSE of POU2AF2/C11orf53 cloned downstream of *luc* gene). Luciferase activity was normalized to Renilla activity. The statistical analysis was done by two-tailed unpaired Student’s t-test, *P < .05, **P < .01.

In agreement with the mechanism of action of previously described USEs [27, 30, 34, 40] and the results of this study, the *DplUSE* RNA sequence appears to act as a platform for the recruitment of RBPs to regulate gene expression. Modifications of the USE sequences may be involved in the deregulation of gene expression in the context of human disease [34]. To investigate the potential impact of the DplUSE sequence in human diseases, we searched for the occurrence of single nucleotide polymorphisms (SNPs) overlapping with the DplUSE sequence (TTGTTTT) at 450 nt upstream of the PAS ATTAAA (Fig. 7A). We found an oncogene containing an SNP associated with human diseases in the DplUSE sequence (Fig. 7B), which has been associated with an increased risk of development of malignant tumour of colon (MTC) (rs3087967; *POU2AF2/C11orf53* [91]). Of note, *POU2AF2/C11orf53* has been recently described to act as a coactivator of POU2F3 to maintain chromatin accessibility and may therefore control genetic programmes [41]. Additionally, it has also been shown that depletion of *POU2AF2/C11orf53* using an *in vivo* xenograft model of small cell lung cancer repressed tumour growth and delayed progression of disease in mice [41], showing the tumorigenic potential of this gene in specific cellular contexts. Importantly, the MTC risk-associated variant (rs3087967) reflects the acquisition of an ectopic DplUSE consensus, compared with the respective non-risk variant. Therefore, in agreement with our results that show increased gene expression in the presence of a DplUSE consensus in the 3′UTR of genes, we asked if the rs3087967 variant, in the context of the 3′UTR of the tumorigenic *POU2AF2/C11orf53* gene, has a higher expression level. To test this, we cloned the 3′UTR of *POU2AF2/C11orf53*, containing the two variants (risk and non-risk) downstream of GFP, under the control of a CMV promoter. Using the Tol2 system, we performed mosaic transgenesis assays in zebrafish and analysed the larvae at 4 dpf. Quantifying the GFP levels in gut cells, we observed that the risk variant showed higher levels of GFP expression, when compared to the non-risk variant (Fig. 7C and D). These results suggest that the acquisition of an ectopic DplUSE in the 3′UTR of the tumorigenic *POU2AF2/C11orf53* might lead to an ectopic expression of this gene and, consequently, contribute to the development of MTC. We then asked if the ectopic DplUSE introduced by the SNP rs3087967 in the 3′UTR of *POU2AF2/C11orf53* increases gene expression in human cells (HEK293). Reporter plasmids with non-risk and risk variants of the *POU2AF2/C11orf53* DplUSE cloned downstream of the *luc* gene were constructed and transfected into HEK293 cells. Results clearly show that the presence of the risk variant DplUSE leads to a ∼25% increase in luciferase activity in comparison to the non-risk variant (Fig. 7E). These results suggest that DplUSE may play an important role in the clinical context.

## Discussion

USEs are characterized as U- and/or pyrimidine-rich *cis*-auxiliary elements capable of modulating mRNA 3′-end formation by acting as an extra platform for the recruitment of *trans*-acting factors, such as RBPs [1, 6, 14, 18, 19, 21, 27, 37, 40, 65, 71, 92] (Table 1). As a result, USEs are considered to be essential to ensure proper control of gene expression and activity, and paradigm elements to regulate 3′ end processing [18] and modulate alternative polyadenylation [15, 93]. These elements were initially described in retroviruses, such as the HIV-1, contributing for the selective use of the PAS at the 3′ end of duplicated viral transcripts [22, 25, 94]. It was proposed that the existence of USEs in HIV-1 revealed the need for a level of regulation that is not normally required by other PAS. However, this concept was challenged when USEs were identified in cellular genes [15, 16, 20, 23, 27, 33] and it is now widely accepted that the general arrangement of mRNA 3′-end formation in eukaryotes contains a USE associated with a PAS [22, 23, 95].

In this work we found that a short motif (DplUSE) located in the USE of the *Drosophila melanogaster polo* gene [15] is also present near PASs in highly divergent vertebrate species, such as zebrafish, mouse, and human. Interestingly, in zebrafish, the DplUSE enrichment near PASs is even more pronounced than in human and mouse, highlighting the conservation of the genomic structure of these regulatory sequences in the zebrafish animal model. Importantly, we show that this motif has conserved functions in gene expression regulation in the fruit fly, zebrafish and humans, and is required to sustain high levels of gene activity in distantly related bilaterian species. This motif therefore appears to participate in a highly conserved mechanism of gene expression regulation. When analysing the molecular machinery that operates at the DplUSE sequence, we found that human HuR, hnRNP C, and PTBP1 bind specifically to this motif *in vitro*, and hnRNPC and HuR *in vivo*. We also demonstrate that HuR and hnRNP C modulate the expression of DplUSE-containing genes in a human cell line by direct binding to DplUSE motif and that, globally, PTBP1 activates distal polyA site selection.

Our results also support that the DplUSE mode of function is not limited to pre-mRNA 3′ end processing, as observed for the fruit fly, as *in vitro* transcribed *GFP-DplUSE* RNA microinjected into the cytoplasm of zebrafish embryos show increased levels of GFP signal compared with the mutant *DplUSE* RNA. In this case, DplUSE thus acts post-transcriptionally, possibly by increasing mRNA stability and/or translation. In agreement with a possible role in the cytoplasm, the RBPs that bind to DplUSE have been shown to shuttle between the nucleus and the cytoplasm and to act at several levels of mRNA biosynthesis. For example, HuR/ELAVL1 is a classic example of a nuclear-cytoplasmic shuttling protein [96] that regulates alternative polyadenylation [97], mRNA stability [98, 99], and translation [99–101]. PTBP1 has also been shown to shuttle between the nucleus and the cytoplasm [102]; in the nucleus it has crucial roles in splicing [64, 103], polyadenylation [71] and modulation of mRNA stability [104], and in the cytoplasm, it has a relevant role in translation [105–108]. hnRNP C, unlike other hnRNPs, was initially thought to be exclusively nuclear [109], modulating the packaging of nascent transcripts and regulating splicing, and influencing mRNA stability [110–112]. However, it has been shown that this RBP can shuttle into the cytoplasm in certain conditions [113, 114] and influence translation [113, 115, 116].

In light of the described roles of these RBPs, in particular PTBP1 and hnRNP C, in the modulation of USE’s function in human cells [30, 34, 40, 71] (Table 1), it is plausible that, in DplUSE-containing genes, they function by modulating 3′ end-processing, mRNA stability, and/or translation, either individually or cooperatively as previously described [99, 101, 104, 107, 113, 117–119].

Our results support a model in which DplUSE and its associated RBPs regulate gene expression through a complex co- and post-transcriptional mechanism that is gene- and subcellular compartment-specific. This regulation probably arises from the capacity of these RBPs to form distinct complexes with DplUSE, their relative abundance, and the presence of additional *cis*-regulatory motifs in the 3′UTR, collectively enabling functions in the nucleus, at the level of pre-mRNA 3′ end processing and/or APA, as well as in the cytoplasm, influencing mRNA stability. Importantly, this intricate mode of action, particularly in physiologically critical genes, may be dismissed in high-throughput analyses in whole-cell preparations, underscoring the necessity of accounting for subcellular localization effects. Notably, the role of the DplUSE motif in gene expression is consistent across species: DplUSE regulates its mRNA levels in *Drosophila*, human cells, and zebrafish, facilitated by the binding of RBPs (PTBP1, hnRNPC, and HuR). These results support the existence of an ancestral mechanism, which increases gene expression, that has important biological roles and is common to highly divergent bilaterians. Indeed, this seems to be the case, since the DplUSE sequence on average shows higher levels of conservation in vertebrates than their respective 3′UTRs, suggesting a selective pressure to keep this motive in the genome. Nonetheless, as only 31 orthologue genes common to zebrafish, mouse, and human have the canonical DplUSE, it is likely that alternative motifs have evolved. Supporting this hypothesis, we observed when analysing the conservation of the DplUSE motif across vertebrate species that some nucleotides are more conserved than others. We also observed that HuR and hnRNPC exhibit significantly higher binding affinity to the canonical DplUSE sequence when compared to non-canonical variant sequences differing by one or two nucleotides, indicating a preference for this motif specificity in RNA–protein interactions.

Similar to other USEs (Table 1), the DplUSE motif seems to be essential in many biological contexts. In *Drosophila* it was demonstrated that its absence dysregulates the cell cycle gene *polo*, causing severe phenotypes in mitosis in neuroblasts and abdominal segments of the adult fly [15]. In the current work we show that using a dominant negative for the molecular players that operate at the DplUSE sequence, causes severe developmental phenotypes in zebrafish embryos, suggesting that DplUSE-controlled genes and the molecular players that operate at the DplUSE are important for vertebrate development. Indeed, the defects observed in zebrafish embryos injected with *DplUSE* and *DplUSEmt* RNAs support the functional relevance of *DplUSE* motifs during development. Nevertheless, embryos injected with *DplUSEmt* showed more defects compared to the not-injected controls, perhaps indicating that microinjection alone has an impact and/or other proteins might bind to regions of the mRNA other than the DplUSE sequence. Overall, the much stronger phenotypic effects observed in *DplUSE*-injected embryos indicate that the consensus motif has a stronger effect on gene regulation than *DplUSEmt*. We also show that depletion of hnRNP C, HuR, and PTBP1, the binding partners of DplUSE, in a human cell line, affects the expression of several genes from the pool of the 31 DplUSE-containing genes that are common to zebrafish, mouse, and humans and that are enriched for association with human diseases such as nervous system diseases, congenital abnormalities, and malignant neoplasms. *EXT2*, for instance, is a glycosyltransferase deregulated in EXT2-related syndrome, where one of the manifestations of the disease is microcephaly [120], a phenotype partially recapitulated in zebrafish embryos microinjected with *DplUSE* RNA. *CLDN12* is another DplUSE-containing gene, which is overexpressed in colorectal carcinomas [121]. These results lead us to postulate that alterations in the DplUSE sequence might contribute to the development of human disease. This is the case of the colorectal cancer-associated SNP rs3087967, located in the 3′UTR of the gene *POU2AF2/C11orf53*, known to have a tumorigenic potential, and for which the mechanistic association to colorectal cancer development has not yet been understood. Here we show that rs3087967 SNP generates an ectopic DplUSE motif that activates gene expression in intestine cells *in vivo*, in zebrafish. Furthermore, we show that this motif also increases gene expression in a human cell line. These findings represent a reasonable mechanistic explanation for the involvement of rs3087967 SNP in colorectal cancer development.

Overall, our study has emphasized the biological importance of the DplUSE motif, identified in *Drosophila*, and that we now show to have a conserved function regulating gene expression across vertebrates. We also show that this motif and the associated conserved mechanism of gene regulation are of extreme importance to understand several human diseases.

## Supplementary Material

gkaf1340_Supplemental_Files

## Data Availability

RNA-seq datasets were deposited to the NCBI Gene Expression Omnibus (GEO; https://www.ncbi.nlm.nih.gov/geo/) under the accession number GSE288026.

## Notes

Present address: Centre de Recherche de Biologie Cellulaire de Montpellier (CRBM), Université de Montpellier, 34293, Montpellier, France
