# Supplementary material for: A conserved 3′UTR short motif regulates gene expression in vertebrates
Source: Nucleic Acids Res. 2026 Jan 8;54(1):gkaf1340. doi: 10.1093/nar/gkaf1340 (PMC12781889; doi:10.1093/nar/gkaf1340)
Supplement: gkaf1340_Supplemental_Files [file gkaf1340_supplemental_files.zip › Supplementary Data.pdf]

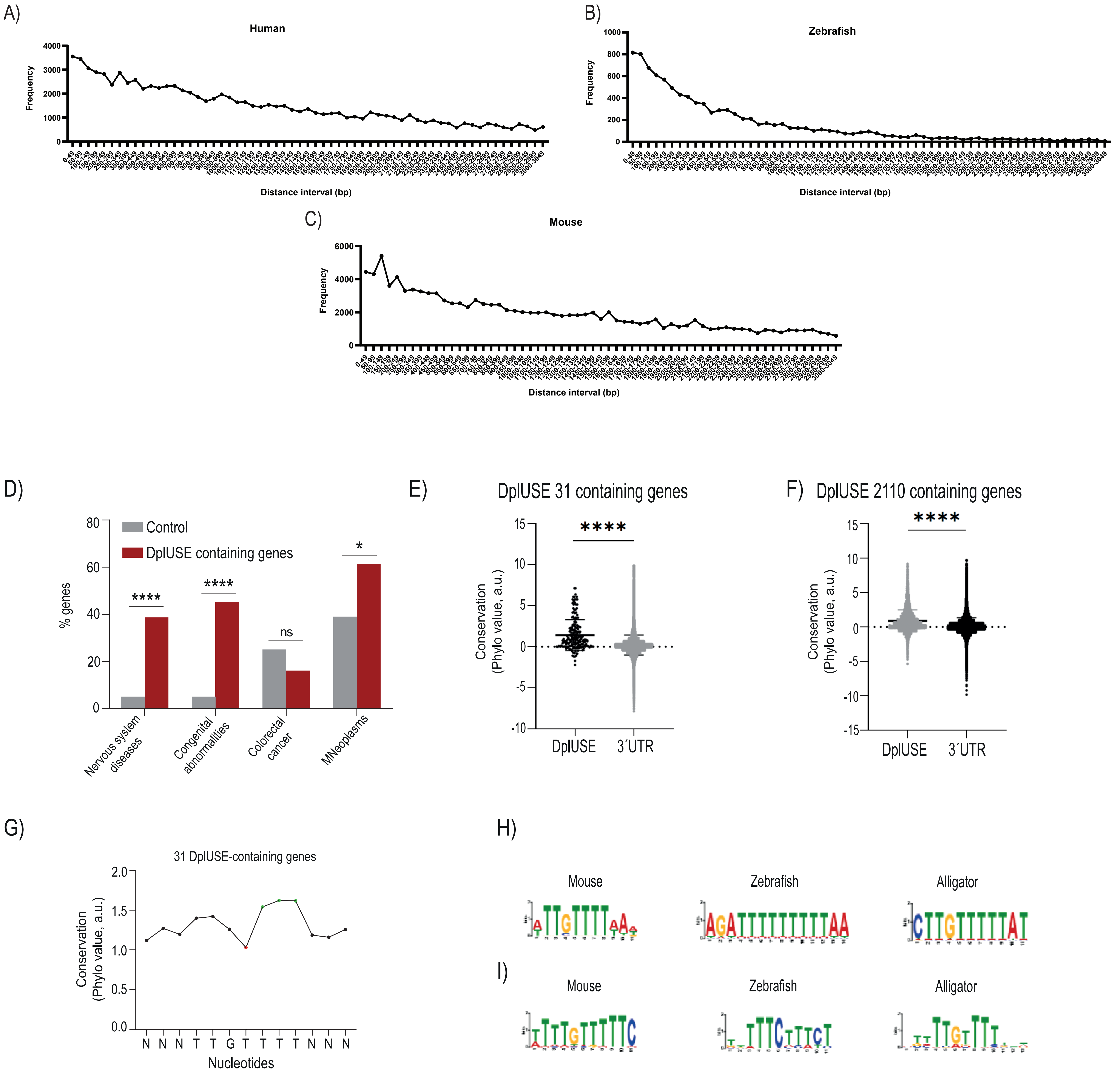

J)

Homo sapiens DpIUSE-containing genes GO Terms	Number of genes	Fold enrichment	FDR
import into nucleous (GO:0051170)	24	2.33	4.53E-02
homophilic cell adhesion via plasma membrane adhesion molecules (GO: 0007156)	35	2.14	1.65E-02
regulation of mRNA processing (GO:0050684)	29	2.13	4.79E-02
regulation of organelle assembly (GO:1902115)	39	2.11	1.12E-02
cell-cell adhesion via plasma-membrane adhesion molecules (GO:0098742)	48	1.9	1.29E-02
positive regulation of cell migration (GO:0030335)	89	1.74	7.50E-04
regulation of Wnt signalling pathway (GO:0030111)	54	1.71	4.42E-02
response to growth factor (GO:0070848)	80	1.69	5.06E-03
regulation of cell migration (GO:0030334)	147	1.67	1.42E-05
positive regulation of cell motility (GO:2000147)	89	1.66	3.29E-03
cell-cell adhesion (GO:0098609)	84	1.66	4.74E-03
cellular response to growth factor stimulus (GO:0071363)	74	1.66	1.24E-02
covalent chromatin modifications (GO:0016569)	59	1.66	4.61E-02
mRNA processing (GO:0006397)	70	1.64	2.52E-02
positive regulation of locomotion (GO:0040017)	90	1.63	5.10E-03

K)

Mus musculus DpIUSE-containing genes GO Terms	Number of genes	Fold enrichment	FDR
multivesicular body sorting pathway (GO:0071985)	11	3.79	3.59E-02
cellular response to glucose stimulus (GO:0071333)	17	3.75	2.28E-03
cellular response to hexose stimulus (GO:0071331)	17	3.62	3.11E-03
cellular response to monosaccharide stimulus(GO:0071326)	17	3.55	3.65E-03
cellular response to carbohydrate stimulus (GO:0071322)	18	3.29	4.74E-03
Golgi to plasma membrane transport (GO:0006893)	14	3.15	3.35E-02
cellular glucose homeostasis (GO:0001678)	21	3.11	2.74E-03
regulation of epithelial to mesenchymal transition (GO:0010717)	22	2.77	6.58E-03
response to glucose (GO:0009749)	22	2.63	1.50E-02
post-Golgi vesicle-mediated transport (GO:0006892)	20	2.6	2.97E-02
response to hexose (GO:0009746)	22	2.55	1.87E-02
response to monosaccharide (GO:0034284)	22	2.52	2.03E-02
response to carbohydrate (GO:0009743)	23	2.32	4.03E-02
vesicle-mediated transport to the plasma membrane (GO:0098876)	23	2.26	4.89E-02
establishment of vesicle localization (GO:0051650)	27	2.23	2.84E-02

L)

Danio rerio DpIUSE-containing genes GO Terms	Number of genes	Fold enrichment	FDR
positive regulation of protein ubiquitination (GO:0031398)	10	6.74	2.80E-03
ribonucleoside metabolic process (GO:0009119)	7	6.44	3.63E-02
positive regulation of protein modification by small protein conjugation or removal (GO:1903322)	10	6.13	4.20E-03
regulation of protein ubiquitination (GO:0031396)	12	5.17	3.28E-03
regulation of protein modification by small protein conugation or removal (GO:1903320)	13	5.06	2.27E-03
cytosolic transport (GO:0016482)	12	3.62	3.62E-02
Notch signalling pathway (GO:0007219)	13	3.25	4.72E-02
endocrine system development (GO:0035270)	18	3.09	1.10E-02
connective tissue development (GO:0061448)	20	2.83	1.29E-02
cartilage development (GO:0051216)	19	2.83	1.79E-02
protein polyubiquitination (GO:0000209)	20	2.56	4.76E-02
organelle fission (GO:0048285)	22	2.5	3.13E-02
small molecule biosynthetic process (GO:0044283)	34	2.21	8.38E-02
mitotic cell cycle process (GO:1903047)	30	2.17	2.60E-02
nucleobase-containing compound biosynthetic process (GO:0034654)	49	2.13	1.37E-03

M)

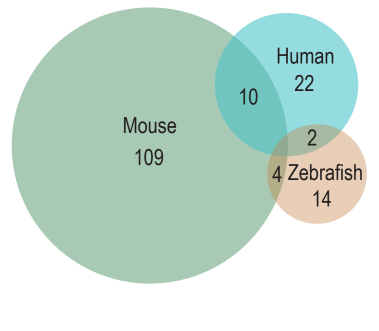

N)

	Human	Mouse	Zebrafish
positive regulation of cell migration (GO:0030335)	•	•	
mRNA processing (GO:0006397)	•	•	
positive regulation of cell motility (GO:2000147)	•	•	
response to growth factor (GO:0070848)	•	•	
positive regulation of locomotion (GO:0040017)	•	•	
cell division (GO:0051301)	•	•	
positive regulation of cellular component movement(GO:0051272)	•	•	
cellular response to growth factor stimulus (GO:0071363)	•	•	
chromatin organization (GO:0006325)	•	•	
mRNA metabolic process (GO:0016071)	•	•	
chromosome organization (GO:0051276)		•	•
establishment of localization in cell (GO:0051649)		•	•
cellular protein localization (GO:0034613)		•	•
cellular macromolecule localization (GO:0070727)		•	•
mitotic cell cycle (GO:0000278)	•		•
mitotic cell cycle process (GO:1903047)	•		•

Figure S1 - (A) Frequency of regions containing the DplUSE sequence (TTGTTTT) and the non-canonical PAS pA1 (ATTAAA) across distance intervals of 50 bp, and up to 3000 bp, in Human, Zebrafish **(B)**, and Mouse **(C)** 3'UTR transcripts. The regions represented in the graph (until 3000 bp distance) correspond to 5329 human genes, 4162 mouse genes and 2599 zebrafish genes. (**D**) Representative graph showing the percentage of the 31 ortholog DplUSE-containing genes that are associated witht the respective disease (DisGenet data source). The control represents the percentage of genes associated with the specific trait/disease in the DisGeNET database. **(E)** Representative graph of the extracted conservation values from each nucleotide within the DplUSE and the 3'UTR sequence, for the 31 ortholog genes (data source: 100 vertebrates). **(F)** Representative graph of the extracted conservation values from each nucleotide within the DplUSE and the 3'UTR sequence, for the 2110 human genes (data source: 100 vertebrates). **(G)** Representative graph showing the average conservation value for each nucleotide within the 31 DplUSE-containing genes. **(H)** Discovered motifs found from the alignment of the converted DplUSEs from the 31 ortholog genes in mouse, zebrafish and american alligator. **(I)** Discovered motifs found from the alignment of the converted DplUSEs from the 2110 human genes in mouse, zebrafish and american alligator. **(J)** DplUSE-containing genes GO terms from human, mouse **(K)** and zebrafish **(L)**. **(M)** Venn diagram representing the common GOs highly enriched between human, zebrafish and mouse (F.E≥1.5). A total of 10 common GO terms were found between human and mouse, 4 between zebrafish and mouse and 2 between human and zebrafish. **(N)** Table representing the GO terms found to be highly enriched in human, zebrafish and mouse.

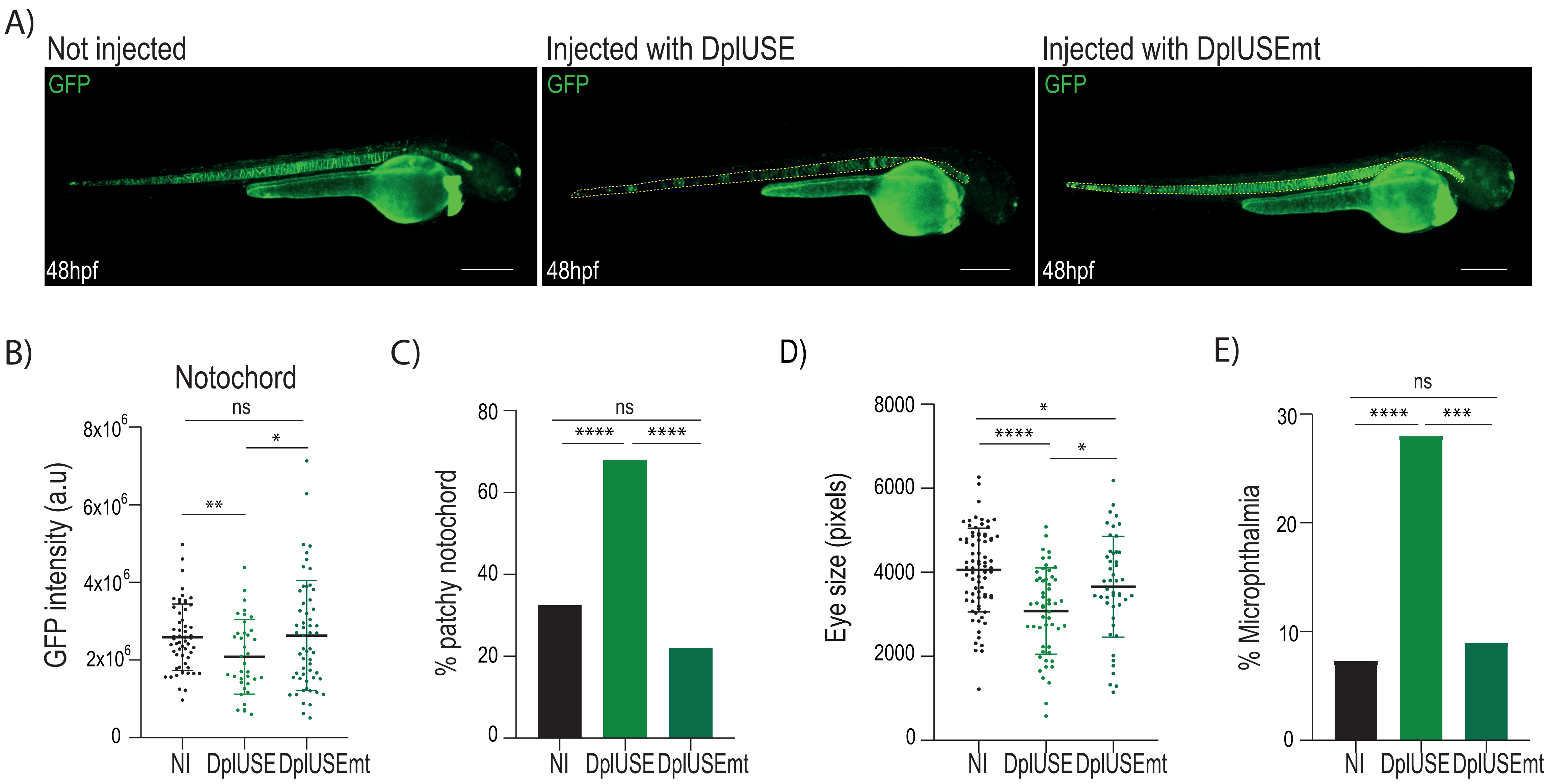

A)
Not injected
Injected with DplUSE
Injected with DplUSEmt
GFP
48hpf
B)
Notochord
GFP intensity (a.u)
8x10^6
6x10^6
4x10^6
2x10^6
0
ns
*
**
NI
DplUSE
DplUSEmt
C)
% patchy notochord
80
60
40
20
0
ns
****
****
D)
Eye size (pixels)
8000
6000
4000
2000
0
*
****
*
E)
% Microphthalmia
30
20
10
0
ns
****
***

Figure S2 - Microinjection of *DplUSE* RNA causes a decrease in GFP expression and microphtalmia in transgenic GFP-DplUSE animals, at 48 hpf. (A) Representative image of GFP expression in transgenic GFP-DplUSE zebrafish embryos not microinjected and microinjected with *DplUSE* RNA or *DplUSEmt*, at 48 hpf. Images were acquired using Leica M205. **(B)** Quantification of GFP expression in notochord (n=57 Not Injected (NI); n=37 DplUSE; n=57 DplUSEmt). **(C)** Quantification of the percentage of transgenic GFP-DplUSE embryos that showed a patchy expression of GFP in the notochord (32,5%, n=99 NI; 68%, n=79 DplUSE; 22%, n=61 DplUSEmt). **(D)** Quantification of the eye size (n=77 NI; n=53 DplUSE; n=47 DplUSEmt) and **(E)** microphthalmia (7%, n=103 NI; 28%, n=83 DplUSE; 9%, n=91 DplUSEmt) of transgenic GFP-DplUSE embryos not microinjected and upon the microinjection of *DplUSE* RNA and *DplUSEmt* RNA, at 48hpf. Statistical significance was determined by χ^2 test with Fisher correction or by two tailed unpaired t-test. ***$p<0.001$; **$p<0.01$; *$p<0.05$; ns: not significant. (Scale bar = 100µm).

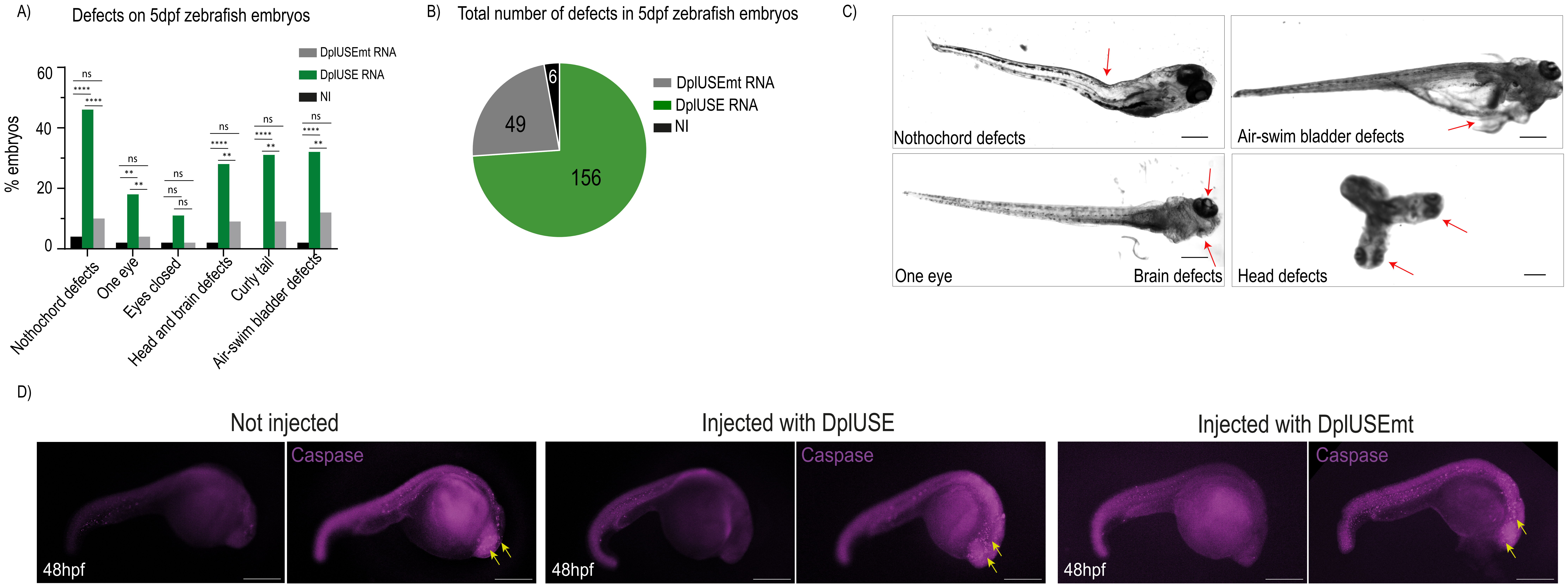
A)
Defects on 5dpf zebrafish embryos
DpIUSEmt RNA
DpIUSE RNA
NI
% embryos
60
40
20
0
ns
****
**
Nothochord defects
One eye
Eyes closed
Head and brain defects
Curly tail
Air-swim bladder defects
B)
Total number of defects in 5dpf zebrafish embryos
6
49
156
DpIUSEmt RNA
DpIUSE RNA
NI
C)
Nothochord defects
Air-swim bladder defects
One eye
Brain defects
Head defects
D)
Not injected
Injected with DpIUSE
Injected with DpIUSEmt
Caspase
48hpf

Figure S3 - Microinjection of *GFP-DplUSE* RNA in one-cell stage embryos leads to the development of various abnormalities 5 days upon the microinjection procedure. (A) Quantification of the several defects developed 5 days in one-cell stage zebrafish embryos not microinjected (in black) or upon the microinjection of *GFP-DplUSE* RNA (in green) and *GFP-DplUSEmt* RNA (in grey). Statistical significance was determined using χ2 with Fisher correction. **(B)** Pie chart representing the total number of defects developed in not microinjected embryos or upon the microinjection of *GFP-DplUSE* RNA and *GFP-DplUSEmt* RNA. **(C)** Representative images showing the various abnormalities upon microinjection of *GFP-DplUSE* RNA at 5 dpf, indicated with red arrows. **(D)** Representative images of embryos stained with anti-cleaved caspase 3 for each phenotypic class (“without cleaved caspase 3” - left and “with cleaved caspase 3”-right) across the three analyzed conditions: not injected (NI), DplUSE-injected, and DplUSEmt-injected embryos. Yellow arrows indicate strong cleaved caspase signal. Scale bar = 100 µm. Statistical significance was determined by χ2 test with Fisher correction or by two-tailed unpaired t-test. ***$p<0.001$; **$p<0.01$; *$p<0.05$; ns $p>0.05$ Images acquired with Leica M205. Scale bar = 100 µm.

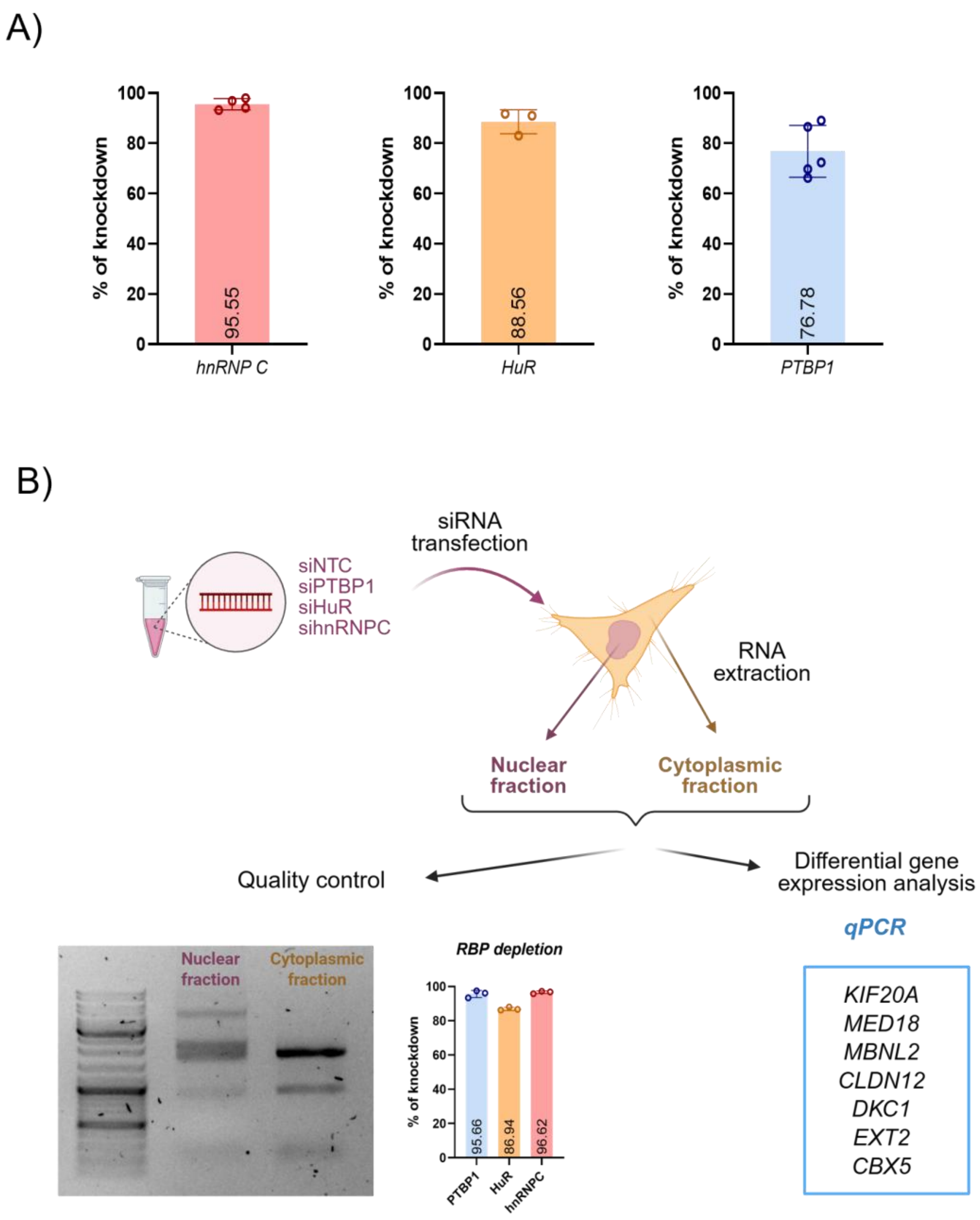

Figure S4 - hnRNP C, HuR and PTBP1 expression was efficiently silenced in HeLa cells. (A) In order to deplete hnRNP C, HuR and PTBP1 expression, HeLa cells were transfected with siRNAs targeting *hnRNPC*, *HuR* and *PTBP1* mRNAs, respectively. A siNTC was also transfected, to act as a negative control. mRNA levels of *hnRNPC*, *HuR* and *PTBP1* were assessed by RT-qPCR, showing a mean depletion, in relation to the control, of around 96% for hnRNP C, 89% for HuR and 77% for PTBP1. **(B)** Schematic representation of the experimental approach for differential gene expression analysis in nuclear and cytoplasmic RNA fractions, showing (left) RNA gel and the enrichment of the precursor ribosomal RNAs in the nuclear fraction and the mean depletion percentage for PTBP1, HuR and hnRNPC, and (right) the analysed genes by RT-qPCR.

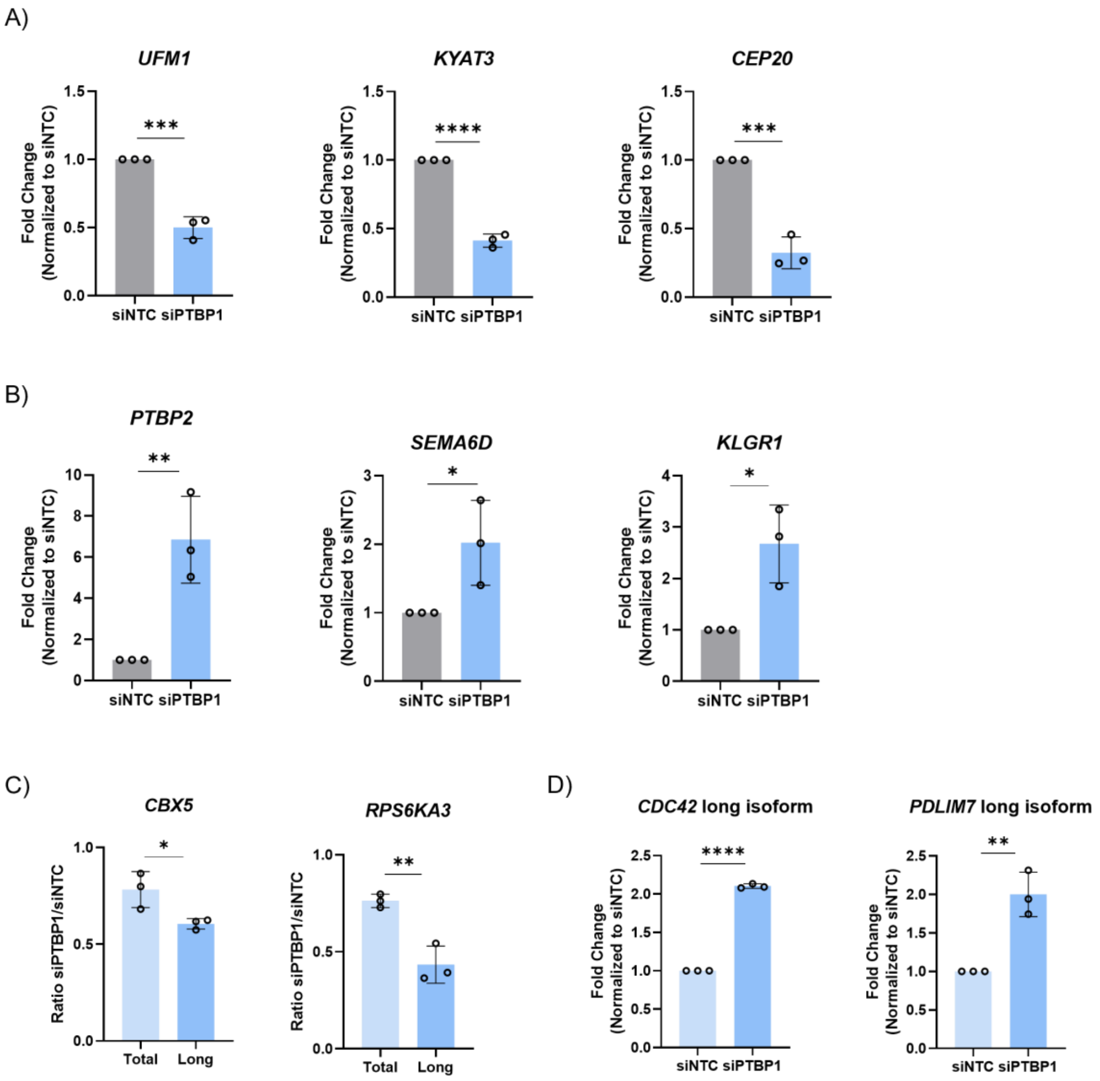

Figure S5 - Validation of RNA-seq data for PTBP1 depletion by RT-qPCR. (A-B) Validation of downregulated **(A)** and upregulated **(B)** genes upon PTBP1 depletion in HeLa cells by RT-qPCR for three independent experiments (n=3). The mRNA levels were normalized to the internal control *18S*, and fold change was then calculated in relation to the siNTC control. **(C-D)** Validation of the APA downregulated **(C)** and IPA upregulated **(D)** genes by RT-qPCR for three independent experiments (n=3). The mRNA levels were normalized to the internal control 18S. **(C)** *CBX5* and *RPS6KA3* mRNA isoform expression levels were measured by RT-qPCR with specific oligonucleotides targeting both mRNA isoforms (total) and the longest mRNA isoform (long). Ratio of the expression levels between the PTBP1-depleted cells and the control (siNTC) is displayed. **(D)** Fold change was calculated in relation to the siNTC control. The data is presented as the mean ± SD of three independent experiments analysed by two-tailed unpaired Student's t-test, where statistical differences are considered when $p<0.05$, and in which $^{**}p <0.01$, $^{***}p < 0.001$ and $^{****}p < 0.001$.

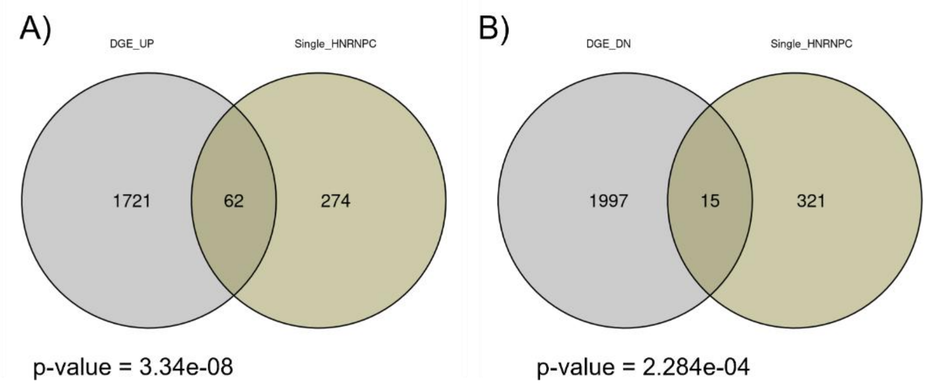

Figure S6. Venn diagram intersections of the human DplUSE-containing genes bound by hnRNP C directly in the DplUSE sequence with the upregulated **(A)** and downregulated **(B)** genes upon hnRNP C depletion in HeLa cells [63]. Data were analyzed using the Fisher exact test, where statistical differences were considered when $p < 0.05$.

Supplementary Table 1 – **(A)** Oligonucleotides, **(B)** antibodies and **(C)** siRNAs used in this study.

A)

Oligonucleotides	Sequence (5'-3')
**FW_useORF**	CCGCTCGAGTAATAAGGCCAAATGACTTCTGGG
**RV_useORF**	CGGGGTACCACAAAGAGCAGCATTGAGG
**FW_useORF_SiteDirectMut**	CCTAAAACAATCTTTGGAATACACC
**RV_useORF_SiteDirectMut**	GGTGTATTCCAAAGATCGTTTTAGG
**InvPCR_3UTRpolo_F1**	ATTAAATGTTTAAACTAAGCAAACG
**InvPCR_3UTRpolo_R**	CTATCACTCGAGAGGCCTTG
**InvPCR_3UTRpolo_F2**	ATTAAATGTTTAAACTAAGCAAACGTGTTTCGAAA
**InvPCR_3UTRpolo_NheI_R**	AATAGCTAGCCTATCACTCGAGAGGCCTTG
**T7_promoter_F**	TAATACGACTCACTATAGGG
**BGH_R**	TAGAAGGCACAGTCGAGG
**KIF20A_F**	GCAATGAACGGGGAATTGGC
**KIF20A_R**	GGATGCCTGTCCCACTTCTG
**CLDN12_F**	TGGCAACCATTGTACTCCCA
**CLDN12_R**	TTCAATGGCAGAGAGGCGAG
**EXT2_F**	GACCTCTGACGAGCTGCAAT
**EXT2_R**	GTCCCAGAGATGCAGACGAC
**MED18_F**	CAAGCCAGCCCATTTGTTCTC
**MED18_R**	TGTCTCCCATTTCTGGCTGTC
**DKC1_F**	TATGTGGGGATTGTCCGGCT
**DKC1_R**	CTGCAGCAATAAGTGGGGGT
**MBNL2_F**	GGCCGTTGTTCGAGAGAGAA
**MBNL2_R**	AAGCATTGCTGCTGCAGTTT
**eGFP_F**	GAGCTGAAGGGCATCGACTT
**eGFP_R**	TTCTGCTTGTCGGCCATGAT
**PTBP1_F**	TCCAGAAGGACCGCAAGATG
**HuR_F**	AGAGAGCGATCAACACGCTG
**HuR_R**	GGAGCCCGCTGATGTACAAG
**hnRNPC_F**	ACAGAGCAAACAAGCAGTAGAGA
**hnRNPC_R**	CACATTAGTCTCATCIIICITCAC
**UFM1_F**	TTCCTTTAAGATCACGCTGACG
**UFM1_R**	GACTGCTGTGAAAGGTGTACTT
**CEP20_F**	GTTCCACGCAAGAAATGGGC
**CEP20_R**	ACCTGTAGTTCCGTTGGACAG
**KYAT3_F**	CTATCAGCCATCCCCGTTTCA
**KYAT3_R**	TTCTTCAGCAGCATCCAGTGT
**KLGR1_F**	TGGCAATAGCTTTGGGGCTT
**KLGR1_R**	GCTGGCACAAGTGGAGTAGT
**PTBP2_F**	ACTGTTCGCTAACACTGGGG
**PTBP2_R**	AGCTTCTTCCACTGTTGCCAT
**SEMA6D_F**	AGGCAAGGCTGTGTATTCCC
**SEMA6D_R**	CCAGTGTTTCTCCAGGACCC
**CBX5_total_F**	ATGAAGCTGACCTGGTTCTTG
**CBX5_total_R**	CTCTTTGTTTTCCGCATCCTCA
**CBX5_long_F**	TGTTCACCCAGCACTAGCAG
**CBX5_long_R**	CAACTCCTGGCTACTTGGCA
**RPS6KA3_total_F**	CAGTCACCAGTTTTGGAACCAG
**RPS6KA3_total_R**	CACAGGGCTGTTGAGGTGA
**RPS6KA3_long_F**	GCCAGAAGCCCAAGATACCAT
**RPS6KA3_long_R**	GGCAGCAATGCCAAGCATC
**CDC42_long_F**	GGTTTTCCTTTGTGCGGTGA
**CDC42_long_R**	CCCACCCCAGACTGACAAAT
**PDLIM7_long_F**	GCACCCCCTTAGTGCCTC
**PDLIM7_long_R**	GGAAGGCAGTGTCAGATGAA

B)

Antibodies
Anti-caspase-3, cleaved (Ab-2) Rabbit pAB (PC679 - Merck)PUREBLUDAPI (1351303- Bio-Rad)

C)

siRNAs	Sequence (5'-3')
**siPTBP1_1**	GCACAGUGUUGAAGAUCAU
**siPTBP1_2**	AACUUCCAUCAUUCCAGAGAA
**siNTC pool**	UGGUUUACAUGUCGACUAA<br>UGGUUUACAUGUUGUGUGA<br>UGGUUUACAUGUUUUCUGA<br>UGGUUUACAUGUUUUCCUA
**siHuR**	UGAACUACGUGACCGCGAAtt
**sihnRNPC**	ACACUCUUGUGGUCAAGAtt
